# Harvest Time Influences Inulin and Phenolic Accumulation of Globe Artichoke (*Cynara cardunculus* L.)

**DOI:** 10.1002/fsn3.72490

**Published:** 2026-10-27

**Authors:** Andrew Tilley, Mark P. McHenry, Julia Anwar McHenry, Yimin Wang, Soudabeh Shemesavar, Malgorzata Kowalczyk, Ruey Leng Loo, Kirsty L. Bayliss, Vicky A. Solah

**Affiliations:** ^1^ Centre for Crop and Food Innovation, Food Futures Institute Murdoch University Perth Western Australia Australia; ^2^ School of Medical, Molecular and Forensic Sciences Murdoch University Perth Western Australia Australia; ^3^ School of Agricultural Sciences Murdoch University Perth Western Australia Australia; ^4^ Mt Lindesay Scotsdale Western Australia Australia; ^5^ Australian National Phenome Centre, Health Futures Institute Murdoch University Perth Western Australia Australia; ^6^ School of Mathematics, Statistics, Chemistry and Physics Murdoch University Perth Western Australia Australia; ^7^ Centre for Computational and Systems Medicine, Health Futures Institute Murdoch University Perth Western Australia Australia

**Keywords:** browning, chlorogenic acid, color, dicaffeoylquinic acid, polyphenol

## Abstract

The influence of harvest time on globe artichoke ( *Cynara cardunculus* L.) inulin and phenolics was investigated to inform selection of superior lines with improved nutritional quality. Lines of unknown genetics were harvested early, middle or late in the growing season. Inflorescence size and edible portion (EP) yield, fructan content, including inulin‐type fructans, inulin profile, phenolic concentrations, color change (browning) after cutting and polyphenol oxidase activity were measured. Early harvested artichokes developed larger inflorescences (average 248.3 g) compared to late harvested artichokes (average 178.5 g) and had higher mean total phenolic concentrations (TPC) (8.67 g kg^−1^) in the EP. Late harvest artichokes had higher mean fructan concentrations (61.56 g kg^−1^ fresh weight) and higher mean yield of EP (54.9%) compared to early harvest artichokes (36.1%). Inulin concentration was positively correlated with mean daily solar radiation exposure, while phenolic concentration was negatively correlated. The CIE b* value (yellowness) of the artichoke receptacle immediately after cutting was significantly, positively correlated with the TPC, chlorogenic acid, and dicaffeoylquinic acid isomer (DiCQA) concentrations. The rate and intensity of polyphenol oxidase‐mediated browning after cutting was correlated with phenolic concentrations, with DiCQA having the strongest association. This research demonstrates the potential benefits of evaluating a wide range of variables to select superior artichoke lines with improved nutritional quality.

## Graphical abstract

Globe artichoke heart fructans were positively correlated with solar radiation exposure while phenolics were negatively correlated. Browning was positively correlated with phenolics, especially dicaffeoylquinic acid isomer. Artichoke hearts with high phenolic contents had higher CIE b* values (yellowness).

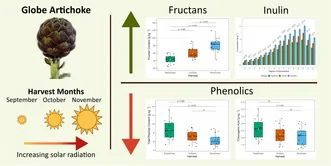

## 1 Introduction

The suitability of globe artichokes ( *Cynara cardunculus* L.) for fresh consumption or value‐add processing is dependent on the quality of the crop. Artichoke quality is determined by its harvest time, size and edible yield, nutritional composition, and susceptibility to polyphenol oxidase (PPO)‐mediated browning (Bonasia et al. 2010; Ghidelli et al. 2013; Pandino, Lombardo, Mauromicale, and Williamson 2011). The size of the artichoke inflorescence is a critical quality attribute for processing as artichoke peeling machines are typically designed to process smaller inflorescences (Bonasia et al. 2010). The edible portion (EP) constitutes only 30% to 40% of the inflorescence and is comprised of the receptacle (heart), inner part of the floral stem (peduncle) and base of the inner bracts. A high EP yield is desired to maximize processing yield and minimize wastage (Bonasia et al. 2010; Pandino, Lombardo, and Mauromicale 2011). Physical attributes, apart from size, contribute to processing suitability although the nutritional composition is also important.

Artichokes are a source of inulin‐type fructans, referred to as inulin. Inulin is a storage carbohydrate primarily located in the cell vacuole and is produced by approximately 15% of all flowering plants (Hendry 1993). Inulin consists of fructose in a linear chain linked by β‐2,1‐glycosidic bonds and terminated with a glucose moiety. The number of fructose units in the chain is defined as the degree of polymerization (DP) and can vary widely within and between species (Márquez‐López et al. 2022). Inulin is usually found in plants as a heterogenous mix of different DPs (Márquez‐López et al. 2022). Inulin consumption may reduce low‐density lipoprotein cholesterol (LDL‐C) levels (Liu et al. 2017), positively affect the human gut, increase colonic absorption of minerals (calcium and magnesium), and increase satiety hormone release (Schaafsma and Slavin 2015). Artichoke receptacles have been reported to contain 29.6–42.6 g kg^−1^ inulin on a fresh weight basis (Pandino, Lombardo, and Mauromicale 2011). Artichoke inulin content and composition can vary widely depending on the cultivar and growing conditions (Livingston et al. 2009).

Artichokes are also an excellent source of phenolic compounds such as hydroxycinnamic acids and flavonoids (Leroy et al. 2010; Wang et al. 2003). Chlorogenic acid (CGA) and dicaffeoylquinic acid (DiCQA) isomers are the predominant hydroxycinnamic acids found in artichoke (Lombardo et al. 2012). Their high concentration potentially contributes to the artichoke's antioxidant and LDL‐C reducing activities (Sahebkar et al. 2018; Salekzamani et al. 2019). Phenolics are nutritionally important but they are also associated with extensive and rapid browning during harvest and processing, which is detrimental to product quality (Cefola et al. 2012; Mishra et al. 2013; Teng et al. 2019).

Browning is primarily driven by PPO catalyzing the oxidation of phenolic compounds that have an *o*‐diphenol group, such as CGA and DiCQA, into *o*‐quinones. The *o*‐quinone groups are highly reactive and can undergo various non‐enzymatic reactions (Ma and Waterhouse 2018) and lead to the formation of large, pigmented polymers that cause the brown color (Cabezas‐Serrano et al. 2009; Teng et al. 2019). Browning can occur within minutes of mechanical damage presenting a major challenge for developing value‐add products (Ghidelli et al. 2015; Pandino et al. 2017; Ricci et al. 2013).

While process design is critical to mitigate browning, susceptibility varies between cultivars with different phenolic concentrations and is influenced by environmental conditions (Cabezas‐Serrano et al. 2009; Pandino et al. 2017) For example, total phenolic content (TPC) was associated with increasing solar radiation (Pandino et al. 2017, 2013) and UV exposure in artichoke leaves (Moglia et al. 2008), upregulating the phenylpropanoid pathway in the exposed tissue. In contrast, artichoke receptacles that had the highest phenolic concentrations were attributed to low average temperatures and increased rainfall (Lombardo, Restuccia, et al. 2015). Cold temperatures may increase phenylalanine ammonia‐lyase (PAL) enzyme activity, a key enzyme in the phenylpropanoid pathway that is responsible for biosynthesis of phenolic acids (Christopoulos and Tsantili 2015; Moura et al. 2010; Sanchez‐Ballesta et al. 2000; Song et al. 2022). Ricci et al. (2013) reported high phenolic concentrations in artichokes correlate with increased antioxidant activity and resulted in lower susceptibility to browning, despite being substrates for browning. Currently, there is no consensus on how harvest time or environmental conditions influence phenolic accumulation and how phenolic concentrations relate to browning susceptibility. An integrated approach that combines these variables is needed to elucidate their respective roles (Tilley et al. 2023).

This study evaluated the influence of harvest time and environmental conditions on edible yield, inulin composition, phenolic composition, PPO activity and PPO‐mediated browning. The aim was to understand how these variables interact to facilitate selection of superior artichoke lines with improved nutritional quality, edible yield and reduced PPO‐mediated browning.

## 2 Materials and Methods

### 2.1 Study Growing Site and Climate

Globe artichokes ( *Cynara cardunculus* L.) were grown at Mt. Lindesay farm, near Denmark, Western Australia (34°51′58.1″S 117°18′04.5″ E), in three distinct fields (field A, B, and C). Fields A and B were adjacent, with Field C located 140 m from Field A and 90 m from Field B. The three fields were used as experimental blocks to control for variation in soil composition, as this could not be analyzed in the study. Daily rainfall was recorded on site for the 28 days prior to each harvest. Climatic data (daily solar radiation, daily maximum and minimum temperature, and total chill hours), for the 28 days prior to each harvest, was sourced from the Australian Bureau of Meteorology and Department of Primary Industries and Regional Development Quickup Dam (DM002) weather station, located 8.7 km south‐east of the trial site (−34° 51′ 58.9032″ S 117° 17′ 56.6052″ E). Chill hours were chosen to represent exposure duration to cold temperatures. Solar radiation data was sourced from the Denmark Agricultural School weather station (34°57′13″ S 117°22′28″ E), located 11.8 km south‐east of the trial site (Bureau of Meteorology 2023) (Department of Primary Industries and Regional Development 2024) (Figure S1).

### 2.2 Management Practices, Inflorescence Harvesting and Yield

Mt. Lindesay farm cultivates artichokes from both self‐propagated seed and offshoots resulting in substantial morphological heterogeneity with no defined variety present. Each artichoke was considered an uncharacterized plant line from the heterogenous population. The artichokes sampled in this study were harvested from plants 2–3 years old with a planting density of 1 m^2^ per plant. Plants were grown using organic management practices, with no fertilizer inputs, weed management, or supplementary irrigation, relying primarily on natural rainfall for water supply.

Globe artichokes were harvested from early to late Spring, with each line harvested once on 25th September, 22nd October, or 21st November 2023, deemed early, mid, and late in the season, respectively. Eighteen inflorescences were randomly harvested at each harvest date, six from each of the three fields. Inflorescences were labeled with the letter A, B, and C indicating the field and the number differentiating individual plants. One primary inflorescence was harvested per plant, with a total of 54 individual plants sampled.

Primary inflorescences were harvested from plants within the principal growth stages 51 to 55 of the Biologische Bundesanstalt, Bundessortenamt, Chemische Industrie scale (Archontoulis et al. 2010), cutting with secateurs 10 cm below the inflorescence with one to two leaves retained. The maximum length and diameter of each inflorescence was measured at harvest using digital calipers (Craftright, Australia) to calculate the L/D ratio (Figure S2) and the approximate volume using the equation for the volume of a cone (Equation (1)), where *D* is the inflorescence diameter divided by two, and *L* is the length of the inflorescence. Samples were transported back to Murdoch University, Western Australia, on ice in Styrofoam boxes and stored in a cool room at 4°C for no more than 48 h.

(1) $$V=\pi D^{2}\frac{L}{3}$$

Prior to preparation, samples were equilibrated to room temperature overnight. Fresh biomass was determined by cutting the floral stem five millimeters below the base of the inflorescence and weighing immediately prior to preparation for testing, to avoid the development of browning at the cut surface.

### 2.3 Sample Preparation

The edible portion (EP) of the inflorescence is composed of the receptacle and the base of the inner bracts. This was prepared by manually removing between 20–30 outer bracts then using a sharp knife to cut off the top 50% of the inflorescence. The EP was weighed, then longitudinally cut into four even quarters. One quarter was immediately used for PPO‐mediated browning analysis (Section 2.4). The remaining three quarters were immediately frozen in liquid nitrogen, then stored at −20°C for a maximum of 2 weeks. The frozen quarters were subsequently combined and lyophilised, ground into a powder in a coffee grinder (Anko, Australia) and passed through a 250 μm sieve. Dried powder samples were stored for a maximum of 4 weeks at −20°C, prior to analysis. The dry weight percentage of the EP was determined by weighing the frozen quarters before and after lyophilisation.

### 2.4 Colourimetric Analysis of Browning Reaction

The color change during PPO‐mediated browning was measured immediately after cutting (Section 2.4). Measurements were taken from 0.5 min to 90 min (0.5, 1, 2, 3, 4, 5, 6, 7, 9, 11, 13, 15, 20, 25, 30, 60, and 90 min) after cutting. At each time point, CIEL*a*b* (L*, a*, and b*) coordinates were generated by taking the average of four measurements (two on each cut surface of the quarter), using a spectro‐guide 45/0 gloss colourimeter (BYK, Germany), set to D65 illuminant. Formulas for calculation of ∆E*, ∆L*, ∆a*, and ∆b* are provided in the Supporting Information (Konica Minolta Sensing Americas Inc., n.d.).

Photographs of browning progression were captured by, photographing EP quarter against a white background at each time point, using a mobile phone camera positioned 10 cm above the sample. The camera settings were f/1.6, ISO 160, focal length 26 mm, exposure time 1/50 s. Photographs were taken under natural light. Photos were cropped depending on the physical size of the sample, to maximize visibility of the cut surface. No other modifications were made to the photos.

Following initial analysis of the colourimetric data, the time points 0.5, 3, 9, and 90 min were selected for further correlation analysis with other variables. The CIE b* value at 0.5 min reflects the yellowness of the edible portion immediately after cutting. Three minutes after cutting for CIE b* and 9 min after cutting for CIE L*, a*, and ∆E* represented time points where the rate of color change decreased. Ninety minutes after cutting for all color parameters reflected the final intensity of color change.

### 2.5 AOAC 999.03 Total Fructan Analysis

Fructan concentration was independently analyzed by Symbio Laboratories Pty. Ltd., Australia, using the AOAC 999.03 measurement of total fructan in foods method (McCleary et al. 2000). Each sample of lyophilised EP powder was analyzed in duplicate and the mean result reported in grams per kilogram on a fresh weight basis.

### 2.6 Inulin Extraction and Analysis

Inulin was extracted from the ground EP samples and analyzed by matrix‐assisted laser desorption ionization—magnetic resonance mass spectrometry (MALDI‐MRMS) as described by Chin et al. (2024) with some modifications. Specifically, each sample eluate was analyzed both undiluted and after a 10 times dilution in ultrapure water to ensure that readings for each DP fell within the calibration range. The inulin concentration (IC) (DP9‐23) was calculated from the sum of concentrations from DP nine to 23, due to internal standards for higher DP's being unavailable. Detailed extraction and analysis methodology are provided in the Supporting Information.

### 2.7 Phenolic Extraction and Analysis

Phenolics were extracted according to Farag et al. (2013) with some modification. Briefly, 1.5 mL of extraction solvent (70% methanol, acidified with 0.1% formic acid) was added to 100 ± 10 mg of the ground EP sample and the mixture vortexed, until the ground EP sample was dispersed. The mixture was sonicated for 30 min, with an excess of ice to prevent heating of the samples (300 W, 40 kHz) (FXP16 Ultrasonic Cleaner, Unisonics Australia). The mixture was then centrifuged at 10,000 *g* for 15 min at 25°C and the supernatant decanted into a 5 mL centrifuge tube. The pellet was extracted twice more under the same conditions and the supernatants pooled. Three replicates of each EP sample were extracted. Extracts were analyzed by Ultra High Performance Liquid Chromatography—Electrospray Ionization—Mass Spectrometry (UHPLC‐ESI‐MS/MS) on the same day and the remaining extract was stored at −70°C until total phenolics analysis (Section 2.8).

To prepare samples for UHPLC‐ESI‐MS/MS analysis, 4 μL of extract was diluted with 996 μL of extraction solvent (1:250) and filtered through a 0.22 μm PTFE hydrophilic syringe filter into a 2 mL screw cap vial with Si/PTFE septa. The filtrate was analyzed immediately.

Chromatographic analysis was performed on a Shimadzu UHPLC Nexera X2 system in conjunction with a Triple Quadrupole (TQ) Mass Spectrometer (LCMS‐8045) equipped with an electrospray ionization interface (ESI), and LabSolutions (Version 5.113, Shimadzu Corporation, Kyoto, Japan) chromatography workstation. UHPLC separation was performed on Waters ACQUITY UPLC BEH C18 column (100 × 2.1 mm, 1.7 μm particle size) at constant temperature of 35°C, with the autosampler tray temperature set up to 15°C. The mobile phase consisted of solution (A) Milli‐Q water—0.1% formic acid (v/v) and (B) acetonitrile. The linear gradient elution was carried at a constant flow rate of 0.5 mL min^−1^ and set as follows: 5% B at 0.0–1.0 min, 5%–95% B at 1–5 min, 95% B at 5–6 min, 95%–5% B at 6–7 min, and 5% B at 7–9 min. The injection volume was 2 μL. The triple quadrupole mass spectrometer was used in negative ionization mode (ESI‐) using multiple reaction monitoring (MRM). Detailed MS/MS operating conditions, MRM transitions and analyte confirmation criteria are provided in Supporting Information.

The concentrations of chlorogenic acid (CGA), cynarin (1,3‐DiCQA), and apigenin‐7‐*O*‐glucuronide (A7G) were calculated from external standard calibration curves. The calibration ranges for each compound were as follows: CGA 0.25–3 mg L^−1^, 1,3‐DiCQA 0.25–3 mg L^−1^, and A7G 0.025–0.5 mg L^−1^. All EP samples were prepared in triplicate. The mean concentrations were calculated from these replicates and expressed as grams per kilogram of fresh edible portion weight (g kg^−1^).

The EP samples showed precursor m/z and MRM transitions consistent with the 1,3‐DiCQA standard, however, the retention time was shifted by approximately 15.25 s and the fragment ion ratio was 57%, compared to 38% for the standard, indicating that the peak does not meet typical confirmation criteria for unambiguous identification of the target analyte. DiCQA detected in the EP samples was not an exact match for the reference standard 1,3‐DiCQA. While the precursor and product m/z were identical in EP samples, the DiCQA peak eluted approximately 15.25 s later than the reference standard. Furthermore, while the fragment ion transitions were the same the fragment ion ratio was 57%.

### 2.8 Total Phenolics Analysis

Total phenolic concentration was estimated using the Folin–Ciocalteu method, adapted to a final volume of 1 mL (Singleton et al. 1999). Detailed methodology are provided in the Supporting Information. The mean TPC was calculated from the three EP extraction replicates and presented as grams of CGA equivalent per kilogram fresh edible portion weight (g kg^−1^). CGA was used to generate the standard curve because hydroxycinnamic acids, such as CGA, are the dominant phenolic class in artichokes (Kyoung Chun and Kim 2004).

### 2.9 Polyphenol Oxidase Extraction and Measurement

Polyphenol oxidase (PPO) extract was prepared using the method described by Mishra, Gautam, and Sharma (2012) with some modifications. 500 ± 20 mg of ground EP sample was mixed with 5 mL of 200 mM sodium phosphate buffer, pH 6.5 at 4°C, containing 30 mM sodium ascorbate, 1% (w/v) polyvinylpolypyrrolidone (PVPP) and 0.5% (v/v) Triton X‐100, then sonicated for 45 min in a water bath with an excess of ice (300 W, 40 kHz) (FXP16 Ultrasonic Cleaner, Unisonics Australia). The mixture was centrifuged at 2132 *g* for 10 min and a 1 mL aliquot of the supernatant was taken for purification. One milliliter of acetone (−20°C) was added drop wise (1:1 v/v) to the aliquot, while mixing with a vortex. The mixture was then centrifuged at 2132 *g* for 10 min and the supernatant discarded. The pelleted precipitate was air dried for 5 min before being mixed with 400 μL of 200 mM sodium phosphate buffer, pH 6.5 at 25°C. The mixture was centrifuged again at 2132 *g* for 10 min and the supernatant decanted and used immediately for analysis. Three replicates of each PPO extraction were performed.

PPO activity was measured using the method described by Ünal and Şener (2016), with some modifications. The assay was carried out using a Cary 60 UV–VIS spectrophotometer (Agilent Technologies Australia, Australia). PPO extract was diluted with 200 mM phosphate buffer, pH 6.5 at 25°C. The dilution factor was sample dependent to ensure the measured change in absorbance was within the assay timeframe. One hundred microliters of the diluted enzyme was pipetted into a 1.5 mL polystyrene cuvette. The spectrophotometer was programmed to monitor absorbance at 390 nm over 60 s with 0.5 s intervals. Recording began simultaneously as 900 μL of freshly prepared 50 mM catechol in 200 mM phosphate buffer, pH 6.5 at 25°C was added to the cuvette. The change in absorbance was monitored over time, and the velocity of the reaction was determined from the initial linear portion of the curve. The linear portion was defined as having a *r* ^2^ of greater than 0.998. One unit of enzyme activity was defined as the amount of enzyme that caused an increase of 0.001 in the absorbance value per minute under the assay conditions. The mean PPO activity was calculated from the three PPO extract replicates and presented as 10^3^ units per gram fresh EP weight (10^3^ U g^−1^). All reactions were carried out in triplicate.

### 2.10 Statistical Analysis

All data analyses were performed using R Statistical Software (R Core Team 2023). Harvest time was the main experimental factor in this study with environmental conditions a consequence induced by harvest time. One‐way ANOVA was used to compare differences in phenolic concentrations (CGA, DiCQA, A7G, and TPC), PPO activity, fructans, IC, and individual inulin DPs between harvests. Prior to analysis the IC and individual DPs were log transformed to meet normality assumption. Tukey's Honestly Significant Difference (HSD) was used where harvest had a significant effect on the dependent variable.

Multiple linear regression was conducted to evaluate the association between the dependent variables CGA, DiCQA, TPC, fructans and IC and the independent variables mean daily solar radiation and total chill hours. Minimum and maximum temperature were excluded from the regression models because minimum temperature was highly collinear with the total chill hours and maximum temperature with daily solar radiation. Where an independent variable was not significant in predicting a dependent variable, it was removed from the model. DiCQA was log transformed to satisfy the normality assumption for the model residuals. Mean values of independent variables were used for analysis of each harvest group.

The change in color parameters after cutting (between harvest groups), was compared by two‐way repeated measures ANOVA. The dependent variables were the color parameters (CIE L*, a*, b*, ∆E*), the between groups variable was harvest time, and the within groups variable was time since cutting. Where significant effects were identified, post hoc pairwise comparisons with Tukey adjustment were conducted. Prior to analysis, the ∆E* and CIE a* variables were log and square transformed due to the data exhibiting a non‐normal distribution and unequal variances, respectively.

To explore correlations between morphological, phenolic and color parameter variables, Spearman's rank‐order correlation coefficients were calculated.

## 3 Results

### 3.1 Inflorescence/EP Weight and Volume

The highest mean inflorescence weight was 248.3 g in September, with sample A5 having the highest weight of 400.8 g (Figure 1A). There was no significant difference between inflorescence weights from September and October and no significant differences in the L/D ratio between harvests (*p* > 0.05) (Figure S2). Mean inflorescence weight in November was 178.5 g, which was significantly lower compared to September (*p* = 0.002) and October (236.2 g) (*p* = 0.012).

**FIGURE 1:**
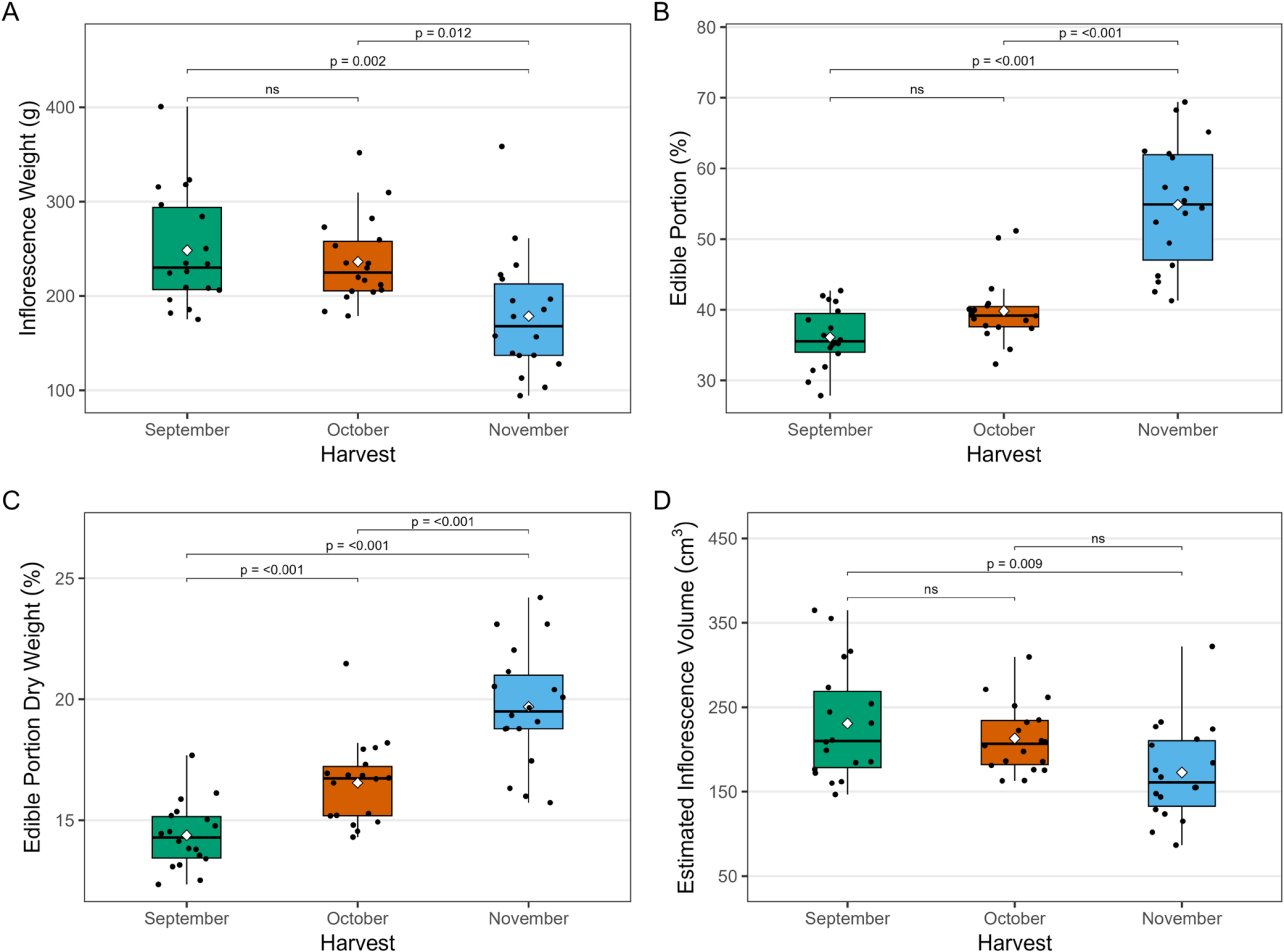
Artichoke inflorescence attributes and edible portion yield grouped by harvest (*n* = 18). (A) Inflorescence fresh weight (g). (B) Edible portion fresh weight as a percentage of inflorescence weight (%). (C) Edible portion dry weight calculated from difference between weight before and after freeze drying (%). (D) Estimated volume of inflorescence (cm^3^). Significance of Tukey HSD post‐hoc test between groups indicated by bars at the top (*p* < 0.05). Boxes show the 25th and 75th quartiles with the median value indicated by the internal black line. The mean value is indicated by the white diamond. The vertical bars identify the maximum and minimum values excluding outliers. Individual data points are presented as black circles.

The EP as a percentage of the inflorescence weight was lowest in September at 36.1%. October was not significantly different to September averaging 39.8% of the inflorescence fresh weight. Artichokes in November had the highest mean EP percentage (54.9%), significantly higher than to September and October harvested artichokes (*p* < 0.001) (Figure 1B, Figure S3). EP dry weight percentage increased with each successive harvest. September had the lowest dry weight percentage of 14.4%, significantly lower than the mean 16.5% in October (*p* < 0.001) and 19.7% in November (*p* < 0.001) (Figure 1C). Estimated inflorescence volume in September harvested artichokes had the highest mean volume of 230.81 cm^3^ (Figure 1D). November harvested artichokes were significantly smaller than September artichokes with a mean volume of 172.57 cm^3^ (*p* = 0.009).

### 3.2 Environmental Factors Affecting Fructan and Inulin (DP9‐23) Concentrations

Fructan content and IC were correlated with daily solar radiation exposure and total chill hours in the 28 d prior to harvest. Total chill hours had a negative correlation with fructan content (*β* = −0.228, *p* < 0.001) and IC (*β* = −0.024, *p* = 0.0024), while mean daily solar radiation exposure was positively correlated with fructan content (*β* = 2.741, *p* = 0.02) and IC (*β* = 0.432, *p* = 0.002) (Figure 2A,B). The models for fructan content (*R* ^2^ = 0.525, *F*(2, 51) = 30.24, *p* < 0.001) and IC (*R* ^2^ = 0.569, *F*(2, 51) = 36.03, *p* < 0.001) were statistically significant explaining 52.5% and 56.9% of the variance, respectively.

**FIGURE 2:**
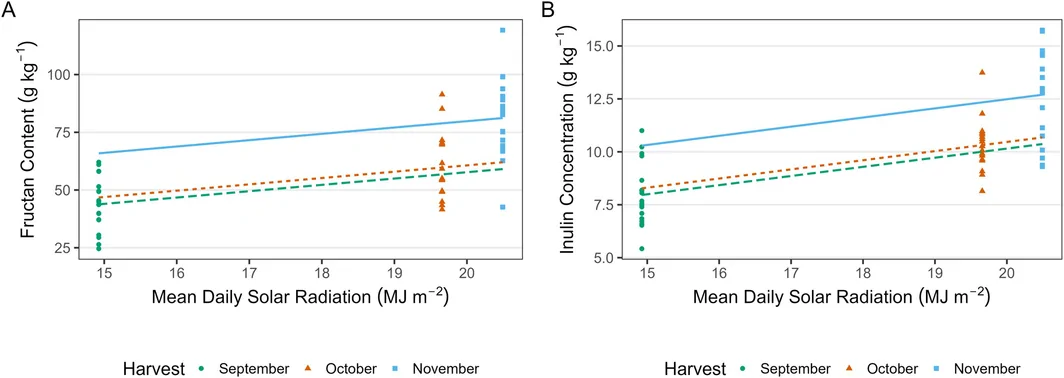
Correlations of fructans and inulin with mean daily solar radiation (MJ m^−2^) 28 days prior to each harvest. (A) Fructan content (g kg^−1^). (B) Inulin concentration (IC) (DP9‐23) (g kg^−1^) Fructan content and IC are presented per kilogram of edible portion fresh weight. The three lines (September: 131 h, October: 118 h, November: 34 h) show the effect of total chill hours on fructan content and IC (*n* = 18). Each line was calculated using the regression equation: Fructan content or IC ~ mean daily solar radiation + total chill hours, where total chill hours is held constant.

### 3.3 Harvest Time Effect on Fructan and Inulin Content

The mean fructan content of artichoke EP in November was 81.16 g kg^−1^ and was significantly higher than September and October harvests (*p* < 0.001) (Figure 3A). October harvested artichokes had a mean fructan content of 59.73 g kg^−1^, which was significantly higher compared to 43.80 g kg^−1^ in September (*p* = 0.005). The fructan content showed substantial variation ranging from 24.59 to 119.23 g kg^−1^, a difference of 485%.

**FIGURE 3:**
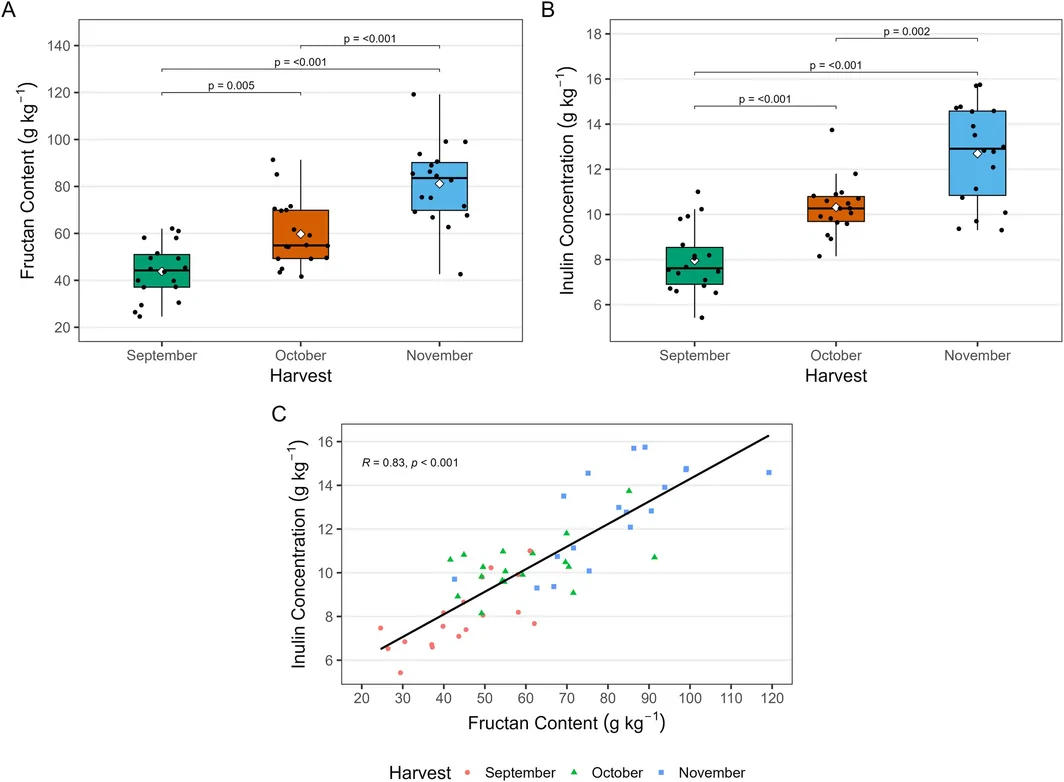
Fructan content and total inulin concentration from artichoke edible portions grouped by harvest (*n* = 18). (A) Fructan content (g kg^−1^). (B) Inulin concentration (IC) (DP9‐23) (g kg^−1^). Fructan content and IC are presented per kilogram of edible portion fresh weight. Significance of Tukey HSD post hoc test between groups indicated by bars at the top (*p* < 0.05). Boxes show the 25th and 75th quartiles with the median value indicated by the internal black line. The mean value is indicated by the white diamond. The vertical bars identify the maximum and minimum values excluding outliers. Individual data points are presented as black circles. (C) Correlation of fructan content with IC (*n* = 54). *R* = Spearman's rank‐order correlation coefficient (*r*_s_), with the corresponding *p* value. Trendlines were generated from linear regression and indicate the direction of association.

Across all samples, IC ranged from 5.42 to 15.75 g kg^−1^, a difference of more than 290% between the highest and lowest ICs. Similar to fructan content, the IC of November harvested artichokes was significantly higher than the September (*p* < 0.001) and October harvests (*p* = 0.004). The IC was significantly higher in October compared to September (*p* < 0.001) (Figure 3B). November had the highest mean IC (12.70 g kg^−1^) with two samples having ICs of greater than 15.5 g kg^−1^. September and October had lower mean IC values of 7.96 and 10.32 g kg^−1^, respectively. The fructan content and IC were not significantly different between field plots (Figures S4 and S5).

Fructan content and IC were positively correlated (*r*_s_(52) = 0.83, *p* < 0.001) (Figure 3C). The artichoke EP with the highest fructan content (120 g kg^−1^) did not have the highest IC. The IC was lower than the fructan content constituting only 18.08%, 17.28%, and 15.64% of fructans for September, October, and November, respectively.

The fructan content was positively correlated with both the EP percentage (*r*_s_(52) = 0.75, *p* < 0.001) and the EP dry weight percentage (*r*_s_(52) = 0.93, *p* < 0.001) (Figure 4A,B). The increase in fructan content and dry weight with each successive harvest (Figures 1 and 3), suggests that the higher dry weight observed in November harvested artichokes is highly associated with the increased fructan accumulation.

**FIGURE 4:**
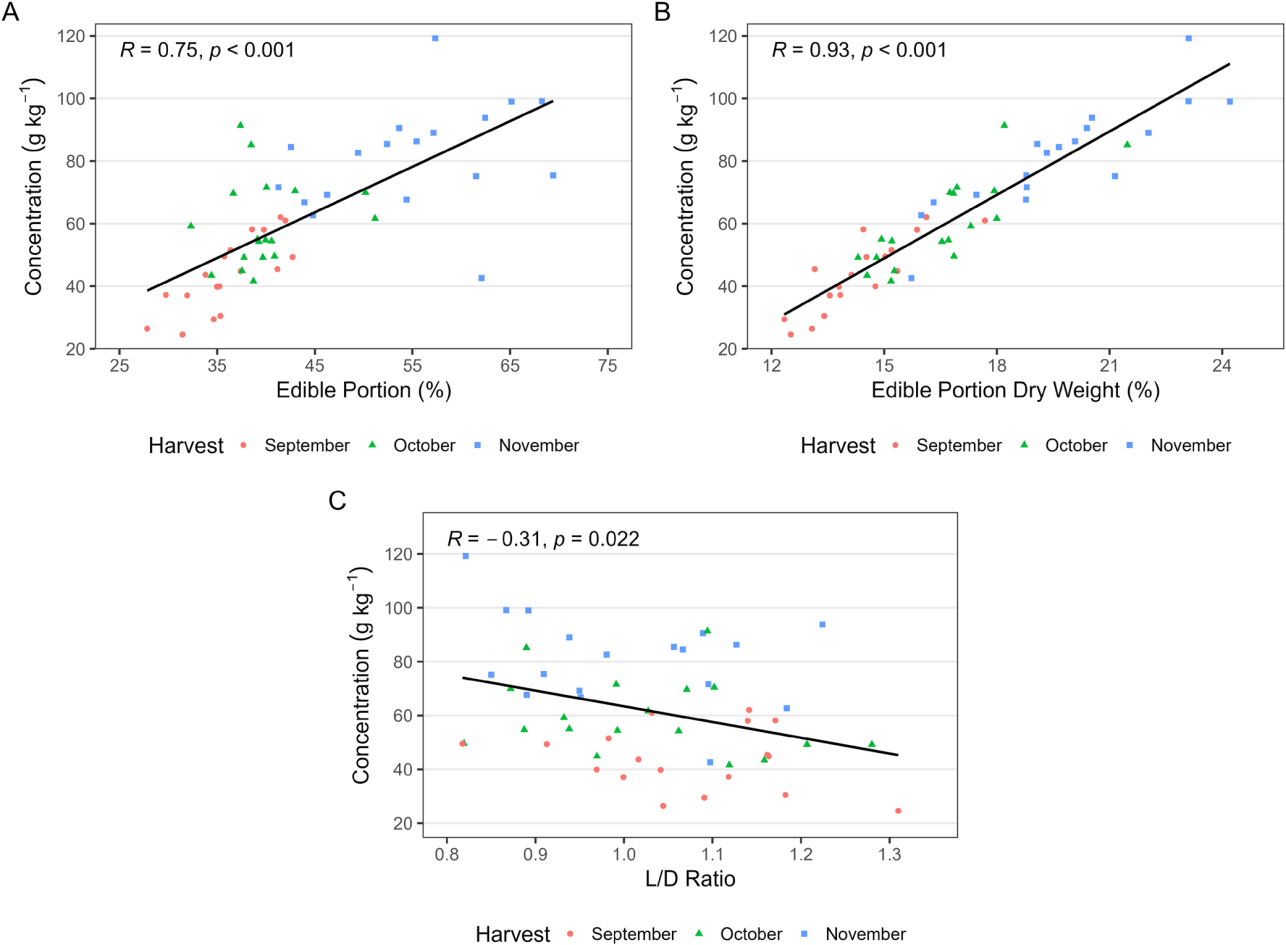
Correlations between fructan concentration (g kg^−1^) and (A) Edible portion (%), (B) Edible portion dry weight (%), and (C) Length/diameter ratio of inflorescence (*n* = 54). Concentrations are presented per kilogram of edible portion fresh weight. *R* = Spearman's rank‐order correlation coefficient, with the corresponding *p*‐value. Trendlines were generated from linear regression and indicate the direction of association.

A weak but significant negative correlation was observed between the L/D ratio and inulin concentration (*r*_s_(52) = −0.31, *p* = 0.022) (Figure 4C). This suggests inflorescence that are more spherical tend to have higher inulin concentrations in the EP.

### 3.4 Inulin DP Profile

Based on the individual DP of inulin measured by MALDI MRMS, a clear increasing trend was observed from early to late harvest (Figure 5, Figures S6 and S7). The abundance of higher DP inulin (DP > 17) increased substantially more than that of lower DP fractions. Specifically, inulin chains with DP17 to DP22 each increased by more than 0.40 g kg^−1^ between September and November (*p* < 0.05). In contrast, inulin with DP9–16 increased by less than 0.33 g kg^−1^ over the same period. Although inulin chains with DP > 23 were detected by MALDI‐MRMS (data not shown), these fractions were not quantified due to the lack of appropriate analytical standards and were therefore excluded from the analysis.

**FIGURE 5:**
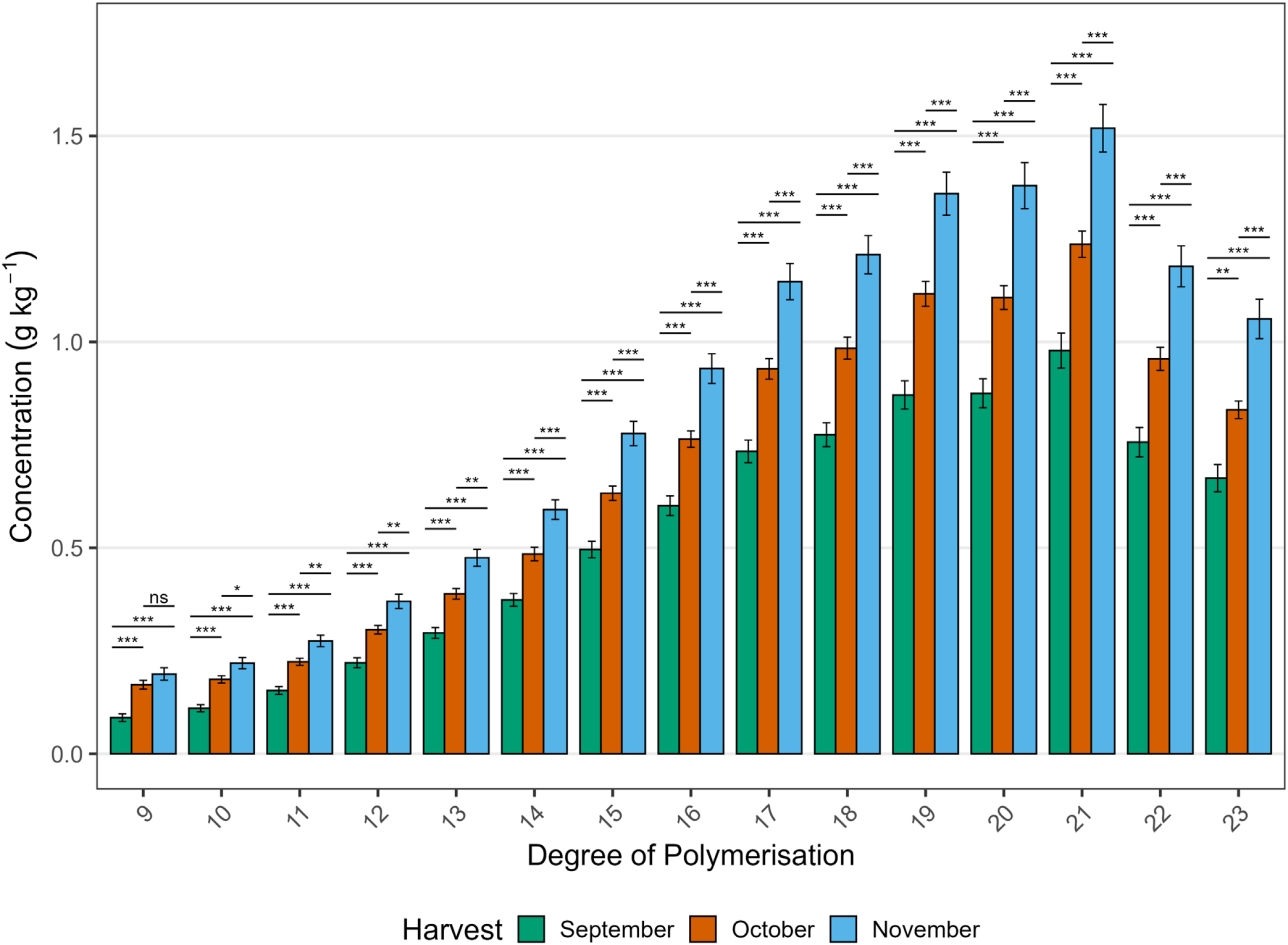
Concentration of individual inulin degrees of polymerization (DP) from artichoke edible portions. Individual DP's are grouped by harvest (*n* = 18). Concentrations are presented per kilogram of edible portion fresh weight. Significance of Tukey HSD post‐hoc test between groups indicated by asterisks, with the groups compared indicated by the relevant bar (^ns^ *p* > 0.05, *p < 0.05, **p < 0.01, ***p < 0.001). Boxes indicate the mean value. The error bars indicate the standard error.

### 3.5 Total Phenolic Content and Solar Radiation Exposure

The TPC had a small but significant correlation with the solar radiation exposure for each harvest. As mean daily solar radiation increased TPC decreased (*R* ^2^ = 0.25, *F*(1, 51) = 9.606, *p* < 0.001) (Figure 6). Solar radiation was not significantly associated with CGA or DiCQA (*p* > 0.05) and total chill hours were not a significant predictor of TPC, CGA, or DiCQA (*p* > 0.05) (data not shown).

**FIGURE 6:**
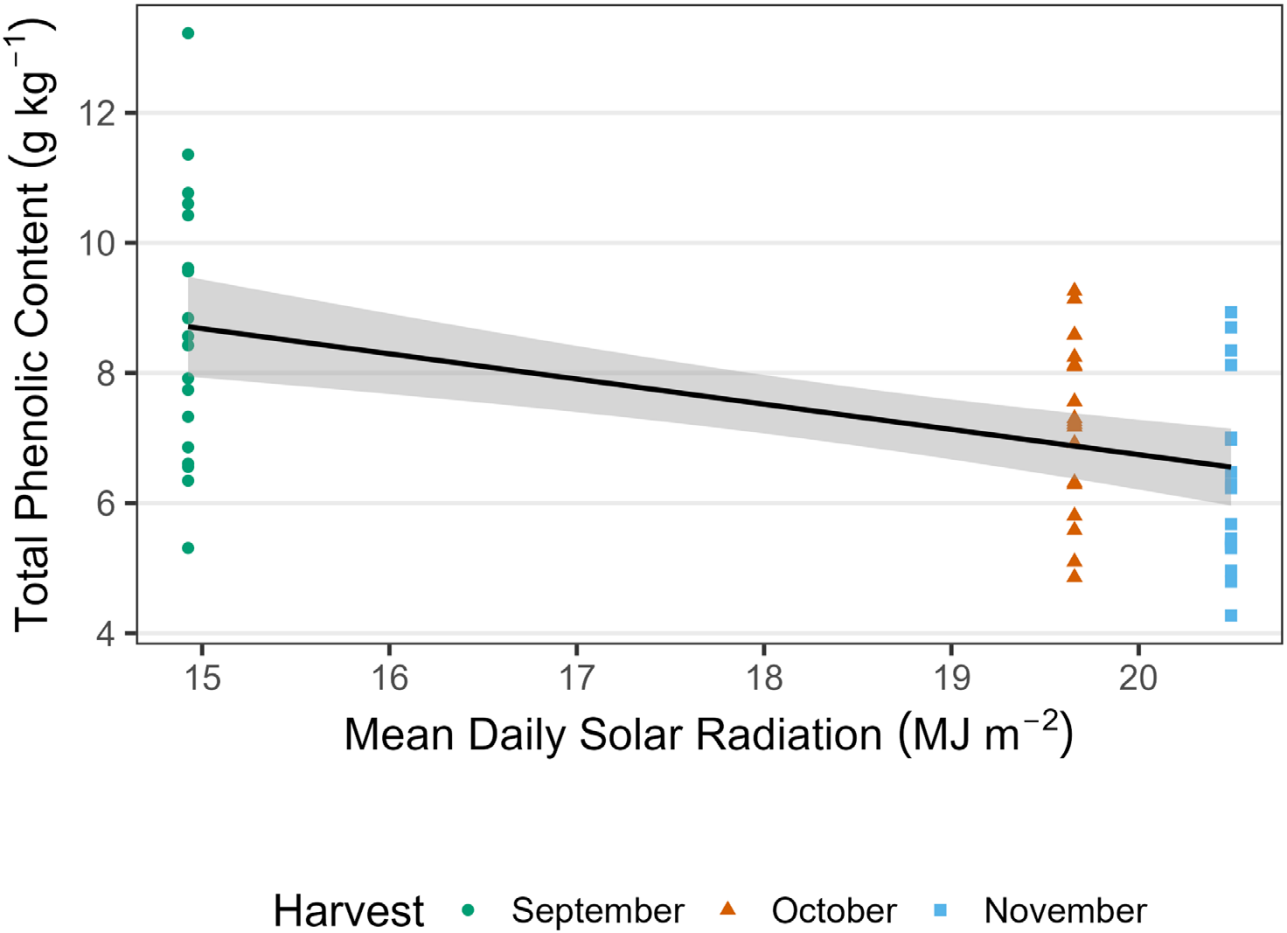
Correlation of total phenolic content (g kg^−1^) with mean daily solar radiation (MJ m^−2^) 28 days prior to each harvest (*n* = 18). TPC is presented as CGA equivalents per kilogram of edible portion fresh weight. The black line was calculated using the regression equation: TPC ~ mean daily solar radiation. 95% confidence intervals are represented by the gray field.

### 3.6 Harvest Time Affects Phenolic Content in Artichoke Edible Portion

The CGA concentration was significantly higher in September harvested artichokes compared to those harvested in November (*p* = 0.034) (Figure 7A). There was no significant difference in CGA concentration between September and October harvests. DiCQA or A7G concentrations were not significantly different in concentration between harvests (Figure 7B,C). Similarly, PPO activity was not significantly different between harvests (Figure S8).

**FIGURE 7:**
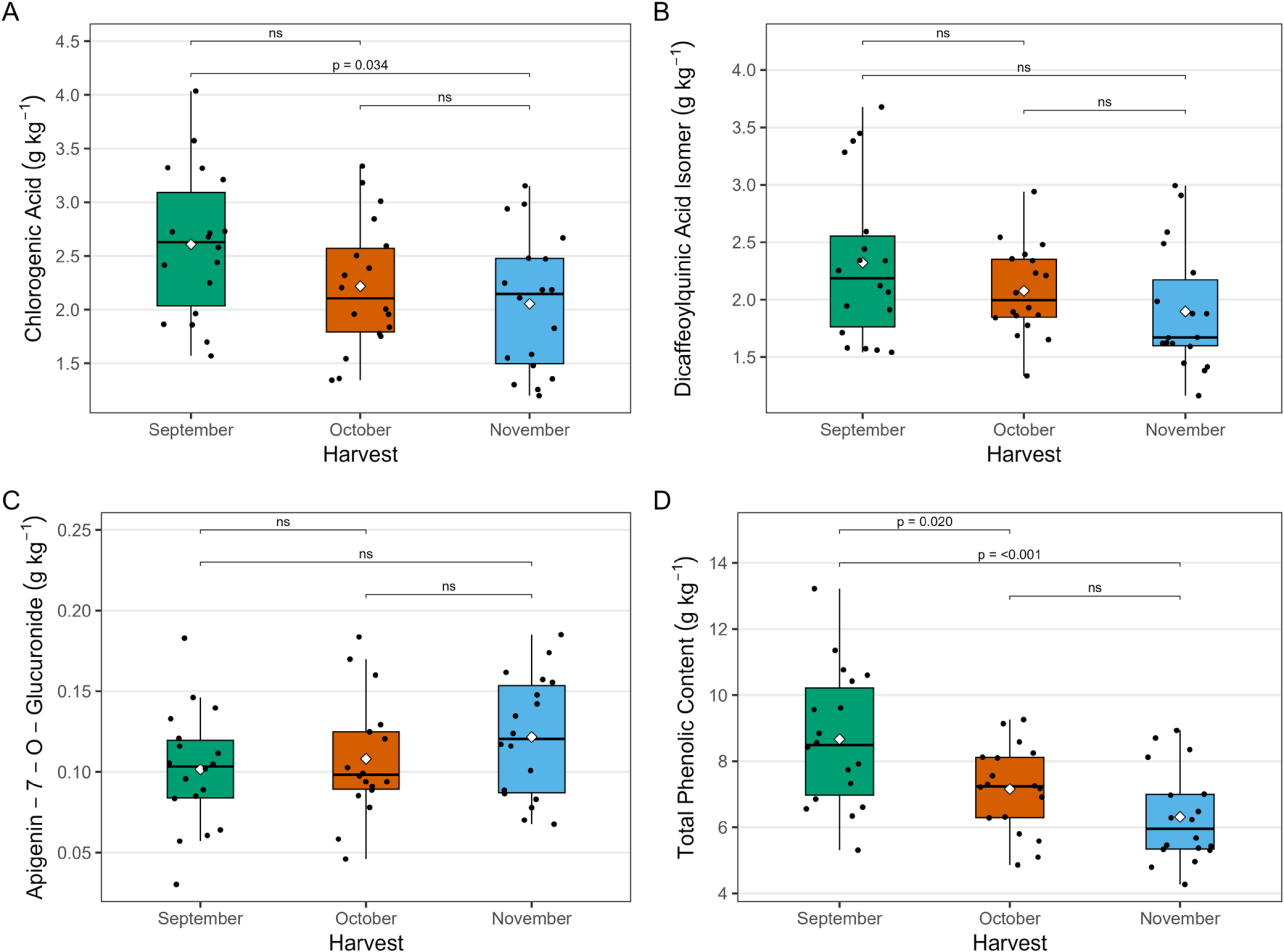
Phenolic concentrations from artichoke edible portions grouped by harvest (*n* = 18). (A) Chlorogenic acid concentration (g kg^−1^). (B) Dicaffeoylquinic acid isomer (g kg^−1^). (C) Apigenin‐7‐*O*‐glucuronide (g kg^−1^). (D) Total phenolic content (g kg^−1^). Concentrations are presented per kilogram of edible portion fresh weight. Total phenolic content is presented as CGA equivalents. Significance of Tukey HSD post hoc test between groups indicated by bars at the top (*p* < 0.05). Boxes show the 25th and 75th quartiles with the median value indicated by the internal black line. The mean value is indicated by the white diamond. The vertical bars identify the maximum and minimum values excluding outliers. Individual data points are presented as black circles.

Significant differences in CGA and DiCQA concentrations were found between samples (Table S1), independent of harvest time. CGA concentration ranged from 1.20–4.04 g kg^−1^ (mean of 2.29 g kg^−1^) and DiCQA ranged from 1.16–3.68 g kg^−1^ (mean of 2.10 g kg^−1^). Two samples exceeded 3.5 g kg^−1^ CGA, and one of these samples exceeded 3.5 g kg^−1^ of DiCQA; both of these samples were collected in September. A7G was detected at lower concentrations compared to CGA and DiCQA (Figure 7C). The A7G concentration ranged from 0.03–0.19 g kg^−1^ (mean of 0.11 g kg^−1^). There were no significant differences in A7G concentration between the harvests (*p* > 0.05).

The TPC of September artichokes was significantly higher than the October (*p* = 0.02) and November harvests (*p* < 0.001). There was no significant difference between October and November harvests (Figure 7D). September had the highest mean TPC of 8.67 g kg^−1^. One sample had an exceptionally high TPC of 13.22 g kg^−1^. October and November had lower mean TPC values of 7.16 and 6.31 g kg^−1^, respectively.

### 3.7 Colourimetric Analysis of PPO‐Mediated Browning

PPO‐mediated browning of artichoke EP was characterized by a rapid initial color change during the first 9 min after cutting, for all color parameters. Measurements were collected from 9 min to 90 min after cutting (Figure 8, Video S1).

**FIGURE 8:**
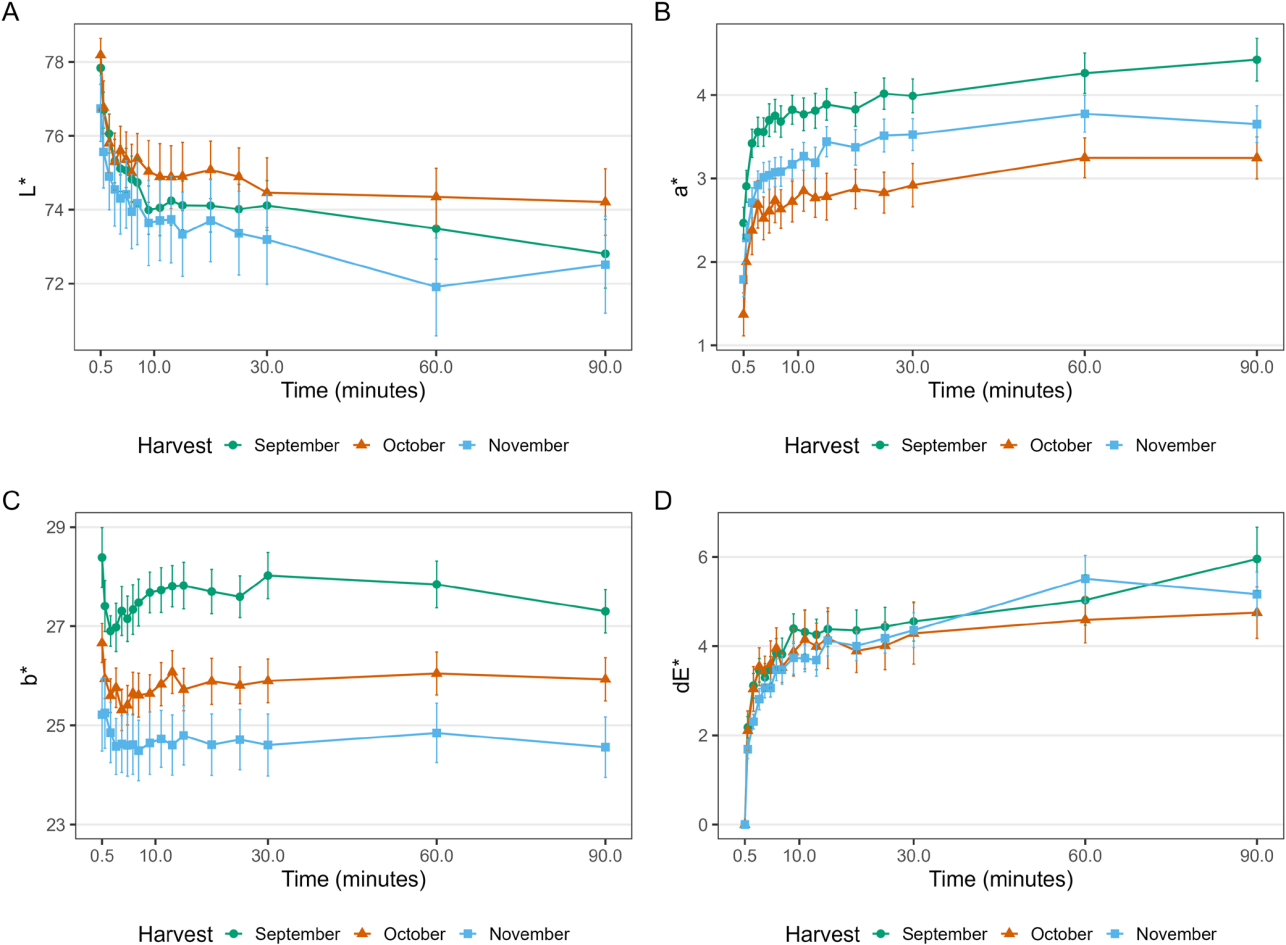
CIE L*a*b* color parameters taken at intervals between 0.5 min and 90 min after cutting the artichoke edible portion. (A) L*, (B) a*, (C) b*, and (D) ∆E* (dE*). Each line represents a harvest (early, mid, and late). Time points are the mean value of samples from that harvest (*n* = 18). Error bars at each time point indicate the standard error.

The CIE L* values decreased significantly during the 90 min after cutting (*p* < 0.001) (Figure 8A). Immediately after cutting (0.5 min) the mean value was 77.59 (range 67.13–82.25) which after 9 min had decreased to a mean value of 74.22 (range 61.58–80.82). From 9 min to 90 min CIE L* continued to decrease, but at a much slower rate (Figure 8A). There were no significant differences in CIE L* or ∆L* values between harvests (*p* > 0.05) (Figure S9A).

CIE a* values increased significantly during the 90 min after cutting (*p* < 0.001) (Figure 8B). For all samples the mean CIE a* value at 0.5 min was 1.88, increasing to 3.24 at 9 min and reaching a mean value of 3.77 at 90 min. The CIE a* values were significantly different between harvests (*p* < 0.001); however, the time*harvest interaction was not significant (*p* > 0.05), and the ∆a* was not significantly different between harvests (*p* > 0.05) (Figure S9B).

The CIE b* values decreased significantly during the 90 min after cutting (*p* < 0.001) (Figure 8C). Within 3 min of cutting, CIE b* values showed a rapid decrease in mean value from 26.75 to 25.77. CIE b* values were significantly different between all harvests (*p* < 0.001); however, the time*harvest interaction was not significant (*p* > 0.05). Mean CIE b* values at 0.5 min were significantly higher in September artichokes compared to November artichokes (*p* = 0.001) (Figure S10), while October samples were not significantly different from either September or November harvests. This finding prompted the inclusion of this parameter in the correlation matrix (Section 3.8). There was no significant difference in the ∆b* values between harvests (*p* > 0.05) (Figure S9C).

The ∆E* represents the total color change between the initial color (0.5 min) and a specific time point. There were no significant differences between harvests (*p* > 0.05) (Figure 8D). All three harvests showed a significant change in color during the first 9 min (*p* < 0.001).

### 3.8 Correlation of Phenolic Content and Edible Portion Color

Concentration of phenolics was correlated with artichoke EP color (Figures S10 and S11). The CIE b* value at 0.5 min after cutting was positively correlated with the concentration of CGA (*r*_s_(52) = 0.44, *p* < 0.001), DiCQA (*r*_s_(52) = 0.59, *p* < 0.001), and TPC (*r*_s_(52) = 0.51, *p* < 0.001), suggesting the yellowness of the edible portion after cutting may increase with increasing phenolic concentrations.

Nine minutes after cutting the rate of color change slowed (Figure 8). Negative correlations were noted between ∆L* at 9 min and DiCQA (*r*_s_(52) = −0.43, *p* = 0.001), A7G (*r*_s_(52) = −0.38, *p* = 0.005), and TPC (*r*_s_(52) = −0.38, *p* = 0.005) (Figure 9, Figure S12), indicating that EP samples with higher CGA, DiCQA, and TPC concentrations had faster initial rates at which CIE L* decreased. Similarly, ∆a* at 9 min was positively correlated with CGA (*r*_s_(52) = 0.4, *p* = 0.003), DiCQA (*r*_s_(52) = 0.39, *p* = 0.004), but not TPC (*p* > 0.05). While ∆E* at 9 min was positively associated with DiCQA (*r*_s_(52) = 0.37, *p* = 0.007), and TPC (*r*_s_(52) = 0.3, *p* = 0.026) (Figure 9, Figure S12).

**FIGURE 9:**
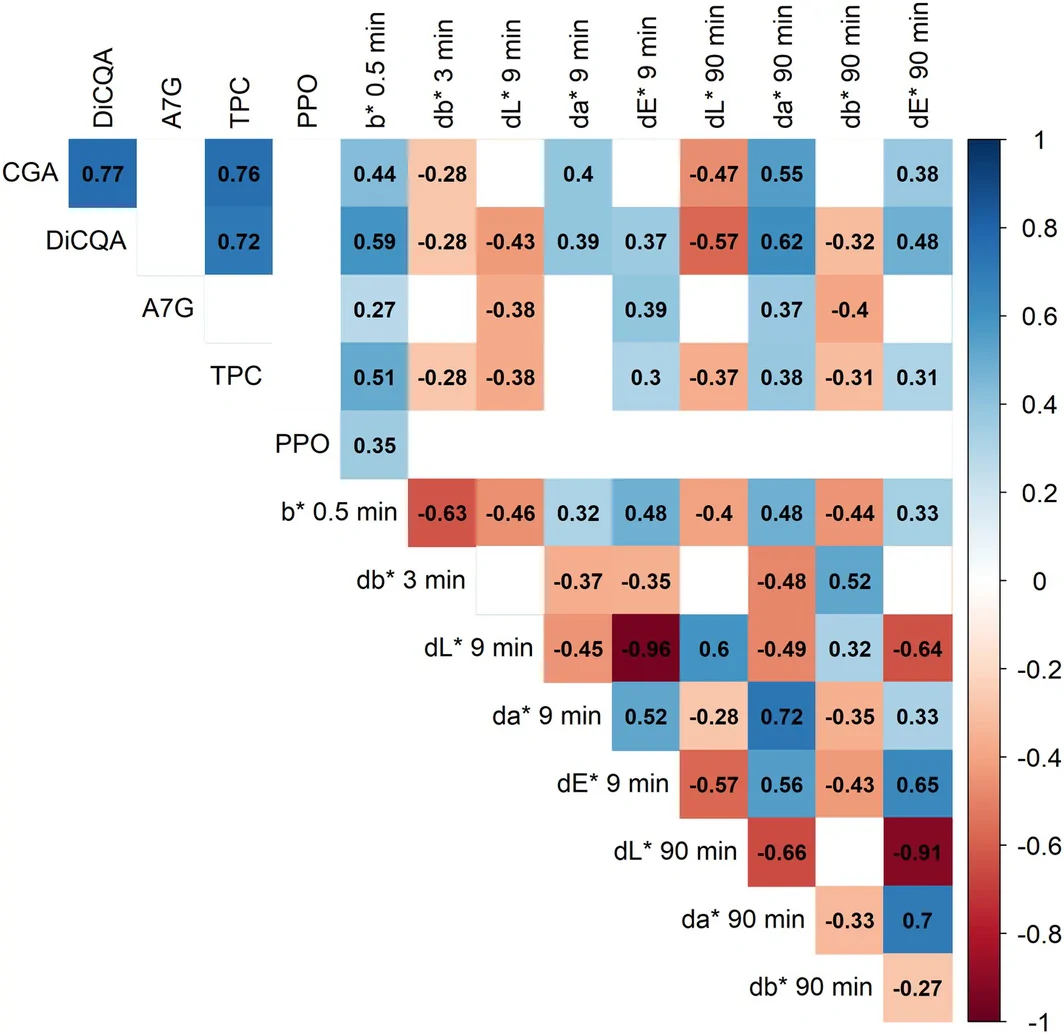
Correlation matrix of color parameters, phenolic concentrations, and PPO activity collected from the total sample population (*n* = 54). Each square represents a Spearman's rank‐order correlation conducted between the variables on the vertical and horizontal axis. Blue colored squares indicate a positive correlation; red squares indicate a negative correlation. The value inside each square is the correlation coefficient (*r*_s_). Squares are only colored if the correlation was significant (*p* < 0.05). A7G, apigenin‐7‐*O*‐glucuronide; CGA, chlorogenic acid; DiCQA, dicaffeoylquinic acid isomer; PPO, polyphenol oxidase activity; TPC, total phenolic content. For CIE L*a*b* variables, d is used to represent ∆.

The measurement collected at the 90 min time point was taken to reflect the final intensity of browning. ∆L* at 90 min was negatively correlated with CGA (*r*_s_(52) = −0.47, *p* = 0.001), DiCQA (*r*_s_(52) = −0.57, *p* < 0.001), and TPC (*r*_s_(52) = −0.37, *p* = 0.006). The ∆a* at 90 min was positively correlated with CGA (*r*_s_(52) = 0.55, *p* < 0.001), DiCQA (*r*_s_(52) = 0.62, *p* < 0.001), A7G (*r*_s_(52) = 0.37, *p* = 0.006), and TPC (*r*_s_(52) = 0.38, *p* = 0.005). This was consistent with CGA, DiCQA, and TPC being positively correlated with ∆E* at 90 min (*p* < 0.05) (Figure 9, Figure S13). There was no correlation between PPO activity and the color parameters (*p* > 0.05).

## 4 Discussion

To the authors' knowledge this study is the first to demonstrate that changes in artichoke quality attributes are correlated with harvest time and environmental conditions, as they changed throughout the season and were interdependent. Both artichoke line and solar radiation were correlated with the fructan content and TPC, which was in turn correlated with inflorescence weight and EP percentage. These results indicate the opportunity to select artichoke lines for specific end products. Early harvest artichokes may be suited to fresh consumption given their larger size, yellow colored EP and high phenolic content, while late harvest artichokes may be suited to value‐add processing due to their higher EP yield, dry matter content and lower phenolic content, which was previously suggested for artichokes produced in Europe (Lombardo et al. 2018).

Inulin accumulation has been shown to require an excess of sucrose in sunflower ( *Helianthus annuus* L.) capitulum, which was dependent on the plants being subject to sufficiently high irradiance (Dosio et al. 2023). During vegetative and flowering phases of artichoke, inulin has been suggested to translocate from the roots to the inflorescence in preparation for flowering (Branca et al. 2022; Raccuia and Melilli 2010). Pandino et al. (2017) observed artichoke EP slices had higher IC from later harvested artichokes, suggesting that this was due to increased solar radiation exposure, although this was not supported with data. The current study provides further evidence that solar radiation is a key factor in inulin accumulation in the artichoke EP.

Fructan analysis indicated exceptionally high concentrations up to 119.23 g kg^−1^ FW (516 g kg^−1^ dry weight (DW) basis). Previous studies have reported concentrations ranging from 109 to 289 g kg^−1^ of DM (Lombardo, Pandino, and Mauromicale 2015; Pandino et al. 2017). The fructan concentrations observed in the current study exceed those previously reported, possibly due to differences in artichoke genotypes (Lombardo et al. 2009). Furthermore, the characterization of individual inulin DPs (DP9–DP23) showed that inulin with larger DPs increased more than smaller DPs between the early and late harvests, confirming that artichoke is a source of long chain inulin (DP > 11) (Heap et al. 2016). Given the potential health benefits of long chain inulin (DP > 11), including increased short chain fatty acid production in the colon and improved intestinal barrier function (Chen et al. 2017; Vogt et al. 2014), the observed increase in long chain inulin may have important nutritional implications.

TPC was negatively correlated with mean daily solar radiation exposure. Solar radiation exposure is generally lower during colder months due to the lower number of daylight hours. Artichoke receptacles harvested in the northern hemisphere's colder months had higher TPC values compared to those harvested in the warmer months (Ricci et al. 2013). This suggests the present findings are consistent with those previously reported. However, other studies have reported that solar radiation, which is composed of the total electromagnetic radiation received, is associated with increased phenolic accumulation in artichoke leaves and tomatoes (Clé et al. 2008; Moglia et al. 2008). In these previous studies the phenolic concentration was measured in the plant part exposed to UV light which forms part of normal daylight. The present study analyzed the EP, which is protected by a layer of outer bracts during plant growth. Bracts contain appreciable concentrations of phenolics and present a physical barrier protecting the EP from direct solar radiation (Lombardo et al. 2010; Pandino, Lombardo, Mauromicale, and Williamson 2011). The progressive decrease in phenolics observed in the EP as solar radiation exposure increased may indicate translocation of phenolics to the outer bracts to provide protection from solar radiation (Lombardo et al. 2010), however further work is needed to validate this hypothesis.

The smaller inflorescence harvested in November may indicate plants are directing resources toward inulin accumulation to prepare for dormancy during the summer period rather than vegetative and inflorescence growth (Raccuia and Melilli 2010). Despite the smaller inflorescence, November artichokes had higher EP yield. Comparing five different varieties harvested at the same time, Bonasia et al. (2010) reported larger inflorescences had higher EP yields due to differences in outer bract thickness. In the current study, we observed November harvested artichokes had thinner bracts compared to artichokes from the earlier harvests. This may reflect a lower moisture content as bracts from these plants were subject to more solar radiation and higher average temperatures, likely leading to greater transpiration. The increase in dry weight was strongly correlated with the increased accumulation of inulin in the artichoke EP. Inulin is the primary driver for increased dry matter content in artichoke roots (Raccuia and Melilli 2010). Despite the smaller inflorescence size, the higher dry weight, inulin concentration and EP yield of late harvested artichokes may present as a higher value product for consumers, given the lower proportion that is inedible and wasted.

The intensity of browning was not influenced by harvest time. Previous work observed that late harvest artichokes exhibited a higher ∆E* compared to early harvested artichokes (Ricci et al. 2013). Genotype dependent differences in the CIE L* values after cutting the artichoke receptacle have been reported previously (Todaro et al. 2010), and were correlated with genotypic differences in biochemical attributes including TPC and PPO activity. The present study noted substantial variation in color change parameters between individual samples when cut. While harvest time was not a determining factor, this variation suggests that selection of low browning susceptible lines is possible.

Several studies have proposed that higher PPO activity is associated with increased PPO‐mediated browning (Brandelli and Lopes 2005; Cefola et al. 2012; González‐Buesa et al. 2011; Mishra et al. 2013; Mishra, Kumar, et al. 2012), but in the present study it was not. PPO in artichoke is important for catalyzing the initial oxidation of *o*‐diphenol groups of phenolics into *o*‐quinones (Tilley et al. 2023). However, oxidized phenolics do not directly yield brown pigments (Murata et al. 2002; Yu et al. 2023), as the intermediary products can lack sufficiently large conjugated double bond systems (Liu et al. 2022). Subsequent slower non‐enzymatic reactions, such as nucleophilic addition, result in polymerization that produces large conjugated double bond systems and browning. While PPO rapidly catalyzes the initial reaction, it is not necessarily the rate limiting factor for browning, nor the determining factor for the intensity of browning (Derardja et al. 2022). It is possible that artichokes sampled in this study had sufficiently high PPO activity that it was not the rate limiting factor of browning. This is supported by the observation that artichoke EP browning proceeds rapidly on the scale of minutes, compared to other plants where browning proceeds over hours to days (Ali et al. 2016; García et al. 2017). Harvest time and the associated environmental factors did not influence artichoke PPO activity, suggesting that the phenolic profile and non‐PPO substrates, that participate in the non‐enzymatic reactions, may be the primary determinants of browning rate and intensity. Further investigation is needed to identify the relative contributions of phenolics and what other molecules participate in browning.

The correlation between CIE b* values after cutting and phenolic concentrations has not been previously reported in globe artichoke. Positive CIE b* values indicate the yellowness of the cut EP surface. Methanolic extracts of artichoke phenolics possessed strong yellow coloration (Figure S14). The concentration of phenolics has a direct influence on the color of artichoke EP and thus its visual quality and agrees with previous research (Cabezas‐Serrano et al. 2009). The correlation between CIE b* values and phenolic concentrations may provide producers with a means to differentiate product quality with consumers and facilitate the development of a rapid, real‐time tool to qualitatively measure the phenolic concentration by in‐field color measurements of EPs. Further work to develop and validate such a tool is needed, and whether similar associations may exist in different food products.

## 5 Conclusion

This study demonstrated that harvest time and environmental conditions influence key quality attributes of globe artichokes. Fructan and inulin concentrations increased in later harvests and were positively associated with solar radiation and negatively associated with chill hours, while total phenolic content declined with increasing solar radiation. Substantial variation between individual artichoke lines indicated that both genetics and environment contribute to these quality traits. Although harvest time did not affect PPO activity or overall browning intensity, phenolic concentrations, particularly DiCQA, were associated with color changes following cutting, suggesting phenolic composition is a more important determinant of browning than PPO activity. The change in quality characteristics with harvest time presents an opportunity to differentiate artichokes based on nutritional attributes, browning susceptibility and yield. This study demonstrates the benefits of evaluating a wide range of variables to differentiate artichoke lines and support selection of lines with superior nutritional quality.

## Author Contributions

**Andrew Tilley:** conceptualization, investigation, methodology, formal analysis, writing – original draft. **Mark P. McHenry:** conceptualization, supervision, writing – review and editing, funding acquisition. **Julia Anwar McHenry:** supervision, writing – review and editing, funding acquisition. **Yimin Wang:** investigation, writing – review and editing. **Soudabeh Shemesavar:** formal analysis, writing – review and editing. **Malgorzata Kowalczyk:** methodology, writing – review and editing. **Ruey Leng Loo:** resources, writing – review and editing. **Kirsty L. Bayliss:** conceptualization, supervision, writing – review and editing. **Vicky A. Solah:** conceptualization, supervision, funding acquisition, writing – review and editing.

## Funding

This work was supported by the Future Food Systems Co‐operative Research Centre (P3‐008) and the Commonwealth through an Australian Government Research Training Program Scholarship [DOI: https://doi.org/10.82133/C42F‐K220].

## Ethics Statement

The authors have nothing to report.

## Conflicts of Interest

The authors declare no conflicts of interest.

## Supporting information


**Figure S1:** Environmental conditions for the 28 days prior to each harvest date. (A) Daily solar radiation (MJ m^−2^), significance of Tukey HSD post hoc test between groups indicated by bars at the top (*p* < 0.05). (B) Maximum and minimum temperatures (°C). Boxes for (A) and (B) show the 25th and 75th quartiles with the median value indicated by the internal black line. The mean value is indicated by the white diamond. The vertical bars identify the maximum and minimum values. (C) Total Rainfall (mL) for the 28 days prior to each harvest date. (D) Total chill hours for the 28 days prior to each harvest date. In all plots, individual data points are presented as black circles.
**Figure S2:** Artichoke inflorescence length/diameter (L/D) ratio grouped by harvest (*n* = 18). Significance of Tukey HSD post hoc test between groups indicated by bars at the top (*p* < 0.05). Boxes show the 25th and 75th quartiles with the median value indicated by the internal black line. The mean value is indicated by the white diamond. The vertical bars identify the maximum and minimum values excluding outliers. Individual data points are presented as black circles.
**Figure S3:** Correlations between (A) Inflorescence weight (g) and edible portion (%), (B) Inflorescence weight (g) and edible portion (g), and (C) Edible portion (%) and edible portion dry weight (%) (*n* = 54). *R* = Spearman's rank‐order correlation coefficient (*r*_s_), with the corresponding *p*‐value. Trendlines were generated from linear regression and indicate the direction of association.
**Figure S4:** Fructan concentration from artichoke edible portions grouped by plot (*n* = 18). Concentrations are presented per kilogram of edible portion fresh weight. Significance of Tukey HSD post hoc test between groups indicated by bars at the top (*p* < 0.05). Boxes show the 25th and 75th quartiles with the median value indicated by the internal black line. The mean value is indicated by the white diamond. The vertical bars identify the maximum and minimum values excluding outliers. Individual data points are presented as black circles.
**Figure S5:** Total inulin concentrations from artichoke edible portions grouped by plot (*n* = 18). Concentrations are presented per kilogram of edible portion fresh weight. Significance of Tukey HSD post hoc test between groups indicated by bars at the top (*p* < 0.05). Boxes show the 25th and 75th quartiles with the median value indicated by the internal black line. The mean value is indicated by the white diamond. The vertical bars identify the maximum and minimum values excluding outliers. Individual data points are presented as black circles.
**Figure S6:** Concentration of individual inulin degrees of polymerization from artichoke edible portions grouped by plot (*n* = 18). Concentrations are presented per kilogram of edible portion fresh weight. Boxes indicate the mean value. The error bars indicate the standard error.
**Figure S7:** Weighted average degree of polymerization from artichoke edible portions grouped by harvest (*n* = 18). Significance of Tukey HSD post hoc test between groups indicated by bars at the top (*p* < 0.05). Boxes show the 25th and 75th quartiles with the median value indicated by the internal black line. The mean value is indicated by the white diamond. The vertical bars identify the maximum and minimum values excluding outliers. Individual data points are presented as black circles.
**Figure S8:** Polyphenol oxidase activity between harvests (*n* = 18). Activity is presented as 10^3^ U g^−1^ of edible portion fresh weight. Significance of Tukey HSD post hoc test between groups indicated by bars at the top (*p* < 0.05). Boxes show the 25th and 75th quartiles with the median value indicated by the internal black line. The mean value is indicated by the white diamond. The vertical bars identify the maximum and minimum values excluding outliers. Individual data points are presented as black circles.
**Table S1:** Fructan and phenolic concentrations, PPO activity, inflorescence & EP and color parameters by sample.
**Figure S9:** The change (∆) in color parameters over time compared to the initial time point (0.5 min). (A) ∆L*, (B) ∆a*, and (C) ∆b*. Color measurements taken at intervals between 0.5 min and 90 min after cutting the artichoke edible portion. Each line represents a harvest (September, October, and November). Time points are the mean value of samples from that harvest (*n* = 18). Error bars at each time point indicate the standard error.
**Figure S10:** CIE b* values measured at 0.5 min compared between harvests (*n* = 18). Significance of Tukey HSD post hoc test between groups indicated by bars above (*p* < 0.05). Boxes show the 25th and 75th quartiles with the median value indicated by the internal black line. The mean value is indicated by the white diamond. The vertical bars identify the maximum and minimum values excluding outliers. Individual data points are presented as black circles.
**Figure S11:** Correlations between CIE b* values at 0.5 min after cutting and phenolics. (A) Chlorogenic acid concentration (g kg^−1^), (B) Dicaffeoylquinic acid isomer (g kg^−1^), and (C) Total phenolic content (g kg^−1^) (*n* = 54). Concentrations are presented per kilogram of edible portion fresh weight. Total phenolic content is presented as CGA equivalents. *R* = Spearman's rank‐order correlation coefficient (*r*_s_), with the corresponding *p* value. Trendlines were generated from linear regression and indicate the direction of association.
**Figure S12:** Correlations of chlorogenic acid concentration (g kg^−1^), dicaffeoylquinic acid isomer (g kg^−1^), and total phenolic content (g kg^−1^) with ∆L*, ∆a*, and ∆E* values at nine min (*n* = 54). Concentrations are presented per kilogram of edible portion fresh weight. Total phenolic content is presented as CGA equivalents. The letter d is used to denote ∆ on the *y*‐axis. *R* = Spearman's rank‐order correlation coefficient (*r*_s_), with the corresponding *p* value.
**Figure 13**. Correlations of chlorogenic acid concentration (g kg^−1^), dicaffeoylquinic acid isomer (g kg^−1^), and total phenolic content (g kg^−1^) with ∆L*, ∆a*, and ∆E* values at 90 min (*n* = 54). Concentrations are presented per kilogram of edible portion fresh weight. Total phenolic content is presented as CGA equivalents. The letter d is used to denote ∆ on the *y*‐axis. *R* = Spearman's rank‐order correlation coefficient (*r*_s_), with the corresponding *p* value.
**Figure S14:** Photo of three phenolic extracts from artichoke edible portions in 70% methanol + 0.1% formic acid. The methanolic extracts exhibit a strong yellow color.

**Video S1:** Screen shot from video collation of PPO‐mediated browning of artichoke quarters taken over 90 min. The graphs from left to right show the change in CIE L*, CIE a*, CIE b*, and CIE ΔE* (dE*). Each line represents a harvest (early, mid, and late). Time points are the mean value of samples from that harvest (*n* = 18). Error bars at each time point indicate the standard error The video can be viewed by double clicking the .mp4 link below.

## Data Availability

The data that support the findings of this study are openly available in Mendeley Data at: Tilley, Andrew (2024), “Globe artichoke ( *Cynara cardunculus* ) phenolic profile influences PPO‐mediated browning—data”, Mendeley Data, V1, doi:10.17632/ncvt97r2jm.1.
