# Supplementary material for: Harvest Time Influences Inulin and Phenolic Accumulation of Globe Artichoke (Cynara cardunculus L.)
Source: Food Sci Nutr. 2026 Oct 27;14(11):e72490. doi: 10.1002/fsn3.72490 (PMC13711686; doi:10.1002/fsn3.72490)
Supplement: Supplementary file 1 [file FSN3-14-e72490-s001.docx]

**Supplementary Information**

**1.0 Methods**

**1.1 Reagents and Standards**

For inulin analysis ultrapure water was used (Milli-Q IQ-7000, Merck KGaA, Darmstadt, Germany). LC-MS grade solvents (acetonitrile, methanol) and porous graphitized carbon solid phase extraction (PGC-SPE) cartridges (HyperSep Hypercarb Column 200 mg in 3 mL tube size) were used for oligosaccharide purification (Thermo Fisher Scientific Australia Pty Ltd., Scoresby, Australia). Inulin-^12^C with DP of 10 (STDDP10, purity of 92.9 %) and 12 (STDDP12, purity of > 90.0 %) (Elicityl SA ,Crolles, France) were used with an inulin-^13^C, a stable isotope internal standard (Cambridge Isotope Laboratories Inc., Tewksbury, MA, USA). The matrix-assisted laser desorption/ionisation (MALDI) matrix, α-cyano-4-hydroxycinnamic acid butylamine salt (CHCA-BA) (Santa Cruz Biotechnology Inc., Campbellfield, Australia). A chicory inulin standard from Sigma-Aldrich (Steinheim, Germany) was used.

For the analysis of phenolics and PPO, reference standards chlorogenic acid (CGA) (PHR2202), cynarin (1,3 dicaffeoylquinic acid) (1,3-DiCQA) (PHL89179), and apigenin-7-*O*-glucuronide (A7G) (04490590), as well as Folin & Ciocalteu’s phenol reagent (F9252) and catechol (135011) were purchased from Sigma-Aldrich (Macquarie Park, NSW, Australia). LC-MS grade acetonitrile (LC015), methanol (34966), and formic acid (56302) as well as analytical grade L-ascorbic acid (AA022), sodium dihydrogen phosphate (SO03310500), and sodium phosphate dibasic (SA026) were sourced from ChemSupply Australia (Gillman, SA, Australia).

**1.2 Formulas for calculation of colour parameters**

∆*E** was calculated for each time point using the formula below (Equation (S1)), where $L_{0}^{*}$, $a_{0}^{*}$, and $b_{0}^{*}$ are the initial values (0.5 min) and $L_{t}^{*}$, $a_{t}^{*}$, and $b_{t}^{*}$ are the values from a subsequent time point (Konica Minolta Sensing Americas Inc., n.d.):

$\text{∆}E_{ab}^{*}= \sqrt{\left( L_{t}^{*}- L_{0}^{*} \right)^{2}+ \left( a_{t}^{*}- a_{0}^{*} \right)^{2}+ \left( b_{t}^{*}- b_{0}^{*} \right)^{2}}$	(S1)

∆*L**, ∆*a**, and ∆*b** were calculated for each time point using formula (Equation S2 – S4):

$\text{∆}L^{*}= L_{0}^{*}- L_{t}^{*}$	(S2)

$\text{∆}a^{*}= a_{0}^{*}- a_{t}^{*}$	(S3)

$\text{∆}b^{*}= b_{0}^{*}- b_{t}^{*}$	(S4)

**1.3 Inulin extraction and analysis**

Immediately prior to solid phase extraction, PGC-SPE columns, installed in a Supelco Visiprep™ 12-port SPE Vacuum Manifold (Sigma-Aldrich, Australia) were conditioned with 3 mL 80 % acetonitrile/water (v/v) followed by 3 mL of ultrapure water. Two mL millilitres of filtered inulin mixture was loaded into the conditioned SPE column, washed with 5 mL of ultrapure water, then eluted with 2 mL of 20 % acetonitrile/water (v/v). Eluates were stored at -80 °C and analysed within two months of extraction.

The frozen eluates were thawed at 20 °C and thoroughly vortexed prior to matrix-assisted laser desorption ionisation – magnetic resonance mass spectrometry (MALDI-MRMS) analysis as previously described with some modifications (Chin et al., 2024). Specifically, each sample eluate was analysed both undiluted and after a ten times dilution in ultrapure water to ensure that readings for each DP fell within the calibration range. After mixing 10 μL of sample eluate with 10 μL of internal standard and 20 μL of MALDI matrix, six spots were prepared for each sample dilution by depositing 2.5 µL onto a MTP 384 ground steel target plate. Automated MRMS data acquisition from 40 accumulative scans was performed over the mass range of 500 – 10,000 m/z. Raw data was processed to extract m/z and signal intensity values using the Bruker DataAnalysis software.

**1.4 UHPLC-ESI-MS/MS operating conditions and MRM transitions**

The triple quadrupole mass spectrometer was operated with following settings: nebulising gas flow 2 L min^-1^, heating gas flow 10 L min^-1^, collision induced dissociation (CID) gas pressure 230 kPa, interface voltage -3 kV, interface temperature 300 °C, and desolvation line (DL) temperature at 526 °C.

The MS-MS analysis conditions were optimised for each compound of interest using the multiple reaction monitoring (MRM) LabSolutions optimization program. One multi-component analysis method was created for simultaneous analysis. For quantification (Q) and identification (q), the following precursor-product ion transitions were monitored: CGA *m*/z 353.2 à 191.0 (Q) and 353.2 à 353.2 (q); 1,3-DiCQA *m*/z 515.2 à 515.2 (Q) and 515.2 à 353.15 (q); A7G *m*/z 445.05 à 445.15 (Q) and 445.05 à 269.05 (q). Fragment ion ratios were used for compound positive identity validation with a tolerance of 20 %.

**1.4 Total phenolic analysis**

One-part Folin-Ciocalteu’s reagent was diluted with 16 parts ultrapure water (1:16). 5 μL of EP extract was diluted with 45 μL of extraction solvent. 850 μL of diluted Folin-Ciocalteu’s reagent (1:16 with ultrapure water) was then added to the diluted EP extract, thoroughly vortexed, and incubated at room temperature. After 1 min and before 8 min, 100 μL of 20 % sodium carbonate solution was added and the mixture vortexed. After two hours incubation at room temperature the absorbance was measured at 760 nm (Cary 60 UV-VIS spectrophotometer, Agilent Technologies Australia). The blank consisted of 50 μL of extraction solvent in place of diluted EP sample and a calibration curve generated using CGA.

**2.0 Results**

**2.1 Environmental conditions during harvest period**

Supplementary Figure 1. Environmental conditions for the 28 d prior to each harvest date. A) Daily solar radiation (MJ m^-2^), significance of Tukey HSD post-hoc test between groups indicated by bars at the top (*p* < 0.05). B) Maximum and minimum temperatures (°C). Boxes for A) and B) show the 25^th^ and 75^th^ quartiles with the median value indicated by the internal black line. The mean value is indicated by the white diamond. The vertical bars identify the maximum and minimum values. C) Total Rainfall (mL) for the 28 d prior to each harvest date. D) Total chill hours for the 28 d prior to each harvest date. In all plots, individual data points are presented as black circles.

**2.2 Length/Diameter (L/D) ratio of inflorescence grouped by harvest**

The L/D ratio which represents the inflorescence shape ranged from 0.82 (spherical inflorescence) to 1.31 (ovoid inflorescence) (Supplementary Table 2).

Supplementary Figure 2. Artichoke inflorescence length/diameter (L/D) ratio grouped by harvest (*n* = 18). Significance of Tukey HSD post-hoc test between groups indicated by bars at the top (*p* < 0.05). Boxes show the 25^th^ and 75^th^ quartiles with the median value indicated by the internal black line. The mean value is indicated by the white diamond. The vertical bars identify the maximum and minimum values excluding outliers. Individual data points are presented as black circles.

**2.3 Correlation between edible portion (EP) and inflorescence parameters**

Smaller inflorescences yielded relatively more EP than larger inflorescences (*r*_s_(52) = 0.4, *p* = 0.003) (Figure 15A). A significant positive correlation between inflorescence weight and EP weight was identified (*r*_s_(52) = 0.7, *p* < 0.001) (Figure 15B). Artichokes from November had the highest EP percentage (Figure 15A). Likewise, September and October harvests had relatively consistent EP percentages regardless of inflorescence weight. This suggests that the inedible portion of the inflorescence (outer bracts and top of inner bracts) are smaller or lighter in weight in smaller inflorescence. The EP percentage was positively correlated with the EP dry weight percentage (*r*_s_(52) = 0.76, *p* < 0.001) (Figure 15C).

Supplementary Figure 3. Correlations between A) Inflorescence weight (g) and edible portion (%), B) Inflorescence weight (g) and edible portion (g) , and C) Edible portion (%) and edible portion dry weight (%) (*n* = 54). *R* = Spearman’s rank-order correlation coefficient (*r*_s_), with the corresponding *p* value. Trendlines were generated from linear regression and indicate the direction of association.

**2.4 Fructan content and total inulin concentration (TIC) of EP grouped by field plot**

Fructans and TIC did not differ significantly between samples from different plots (*p* > 0.05) (Supplementary Figures 4 and 5).

Supplementary Figure 4. Fructan concentration from artichoke edible portions grouped by plot (*n* = 18). Concentrations are presented per kilogram of edible portion fresh weight. Significance of Tukey HSD post-hoc test between groups indicated by bars at the top (*p* < 0.05). Boxes show the 25^th^ and 75^th^ quartiles with the median value indicated by the internal black line. The mean value is indicated by the white diamond. The vertical bars identify the maximum and minimum values excluding outliers. Individual data points are presented as black circles.

Supplementary Figure 5. Total inulin concentrations from artichoke edible portions grouped by plot (*n* = 18). Concentrations are presented per kilogram of edible portion fresh weight. Significance of Tukey HSD post-hoc test between groups indicated by bars at the top (*p* < 0.05). Boxes show the 25^th^ and 75^th^ quartiles with the median value indicated by the internal black line. The mean value is indicated by the white diamond. The vertical bars identify the maximum and minimum values excluding outliers. Individual data points are presented as black circles.

**2.5 Inulin DP profile of EP grouped by field plot**

There were no significant differences in concentration of specific DPs between plots (*p* > 0.05) (Supplementary Figure 6).

Supplementary Figure 6. Concentration of individual inulin degrees of polymerisation from artichoke edible portions grouped by plot (*n* = 18). Concentrations are presented per kilogram of edible portion fresh weight. Boxes indicate the mean value. The error bars indicate the standard error.

**2.6 Effect of harvest on inulin weighted average DP**

The weighted average DP (WADP) from DP9 to 23 was significantly higher in September artichokes (*p* = 0.007) (Figure 7). The September samples had a WADP of 18.16, higher than the WADP of samples harvested in October (18.05) and November (18.08).

Supplementary Figure 7. Weighted average degree of polymerisation from artichoke edible portions grouped by harvest (*n* = 18). Significance of Tukey HSD post-hoc test between groups indicated by bars at the top (*p* < 0.05). Boxes show the 25^th^ and 75^th^ quartiles with the median value indicated by the internal black line. The mean value is indicated by the white diamond. The vertical bars identify the maximum and minimum values excluding outliers. Individual data points are presented as black circles.

**2.7 Polyphenol oxidase (PPO) activity of EP grouped by harvest**

Supplementary Figure 8. Polyphenol oxidase activity between harvests (*n* = 18). Activity is presented as 10^3^ U g^-1^ of edible portion fresh weight. Significance of Tukey HSD post-hoc test between groups indicated by bars at the top (p < 0.05). Boxes show the 25^th^ and 75^th^ quartiles with the median value indicated by the internal black line. The mean value is indicated by the white diamond. The vertical bars identify the maximum and minimum values excluding outliers. Individual data points are presented as black circles.

Supplementary Table 1. Fructan and phenolic concentrations, PPO activity, inflorescence & EP and colour parameters by sample.

| **Sample** | **Harvest** | **Fructan** | **TPC** | **CGA** | **DiCQA** | **A7G** | **PPO** | **Weight** | **EP%** | **∆*L** 9 min** | **∆*L** 90 min** | **∆*a** 9 min** | **∆*a** 90 min** | **∆*E** 9 min** | **∆*E** 90 min** |
| --- | --- | --- | --- | --- | --- | --- | --- | --- | --- | --- | --- | --- | --- | --- | --- |
| A8 | S | 26.41 | 13.22 | 3.32 | 3.38 | 0.13 | 925.21 | 195.85 | 27.83 | -5.08 | -8.36 | 2.31 | 3.77 | 6.75 | 9.76 |
| C16 | S | 37.20 | 11.36 | 4.04 | 3.68 | 0.08 | 1348.77 | 175.07 | 29.75 | -4.62 | -5.25 | 1.79 | 3.07 | 5.53 | 6.57 |
| B12 | S | 30.49 | 10.77 | 2.68 | 2.34 | 0.08 | 635.68 | 206.19 | 35.32 | -2.07 | -3.17 | 1.55 | 1.99 | 2.85 | 3.53 |
| A7 | S | 24.59 | 10.60 | 3.21 | 2.25 | 0.03 | 895.67 | 250.09 | 31.44 | -3 | -3.72 | 1.45 | 1.66 | 3.41 | 3.55 |
| C17 | S | 43.65 | 10.42 | 2.71 | 2.59 | 0.11 | 760.79 | 323.04 | 33.8 | -2.66 | -0.69 | 1.24 | 1.36 | 3.05 | 2.56 |
| C19 | S | 37.06 | 9.61 | 3.57 | 3.28 | 0.14 | 2600.39 | 224.26 | 31.93 | -4.97 | -8.48 | 1.83 | 3.47 | 5.82 | 10.01 |
| B13 | S | 60.99 | 9.56 | 3.32 | 3.45 | 0.06 | 426.04 | 208.91 | 41.99 | -3.24 | -8.94 | 1.05 | 2.98 | 3.72 | 9.46 |
| A5 | S | 51.5 | 8.84 | 2.44 | 2.07 | 0.10 | 8.34 | 400.75 | 36.37 | -4.65 | -4.02 | 1.49 | 1.83 | 4.89 | 4.43 |
| A9 | S | 44.83 | 8.56 | 1.96 | 2.12 | 0.12 | 900.26 | 296.77 | 37.41 | -3.09 | -9.47 | 0.61 | 1.39 | 3.2 | 9.69 |
| A10 | S | 49.32 | 8.42 | 2.73 | 1.54 | 0.10 | 755.89 | 208.17 | 42.72 | -3.71 | -6.96 | 1.32 | 2.27 | 4.07 | 7.53 |
| C18 | S | 62.06 | 7.92 | 1.86 | 1.58 | 0.10 | 2043.25 | 185.5 | 41.48 | -4.92 | -0.3 | 1.65 | 0.24 | 5.21 | 1.57 |
| A6 | S | 49.52 | 7.74 | 1.70 | 1.71 | 0.15 | 1915.87 | 318.03 | 35.73 | -4.54 | -2.46 | 0.08 | -0.61 | 5.36 | 4.59 |
| B14 | S | 39.94 | 7.33 | 2.41 | 2.34 | 0.18 | 1187.42 | 234.65 | 35.22 | -5.93 | -6.31 | 1.22 | 2.84 | 6.05 | 6.93 |
| B15 | S | 45.42 | 6.86 | 1.86 | 1.57 | 0.11 | 322.08 | 284.19 | 41.17 | -1.99 | 1.06 | 1.18 | 1.43 | 2.58 | 3.33 |
| C15 | S | 29.43 | 6.61 | 2.72 | 1.91 | 0.09 | 2015.27 | 181.87 | 34.65 | -3.92 | -4.63 | 1.61 | 1.66 | 4.32 | 5.14 |
| B17 | S | 58.03 | 6.56 | 2.58 | 2.44 | 0.06 | 1420.50 | 233.85 | 39.79 | -5.29 | -10.77 | 1.82 | 3.14 | 5.9 | 11.37 |
| B16 | S | 39.80 | 6.34 | 1.57 | 1.95 | 0.12 | 748.67 | 315.57 | 34.98 | -4.37 | -1.92 | 1.59 | 1.56 | 4.68 | 4.05 |
| C14 | S | 58.15 | 5.31 | 2.25 | 1.56 | 0.06 | 471.50 | 225.97 | 38.58 | -1.25 | -0.16 | 0.64 | 1.18 | 1.66 | 3.16 |
| B23 | O | 49.20 | 9.26 | 3.34 | 2.94 | 0.12 | 650.99 | 178.72 | 37.75 | -2.45 | -5.49 | 1.49 | 2.86 | 2.91 | 6.2 |
| C21 | O | 71.57 | 9.14 | 2.85 | 2.39 | 0.10 | 1102.10 | 219.92 | 40.06 | -1.89 | -2.29 | 1.05 | 0.95 | 2.19 | 2.5 |
| B20 | O | 43.41 | 8.59 | 2.59 | 2.06 | 0.12 | 538.12 | 206.28 | 34.41 | -2.59 | -2.62 | 1.5 | 1.73 | 3.01 | 3.19 |
| A15 | O | 54.72 | 8.24 | 3.01 | 2.34 | 0.16 | 1000.97 | 204.11 | 39.97 | -3.48 | -5.46 | 1.36 | 2.41 | 4.29 | 6.22 |
| A11 | O | 61.64 | 8.12 | 1.54 | 1.65 | 0.18 | 326.54 | 198.81 | 51.17 | -3.74 | -1.59 | 1.29 | 1.89 | 4.12 | 3.68 |
| A12 | O | 54.24 | 8.10 | 1.96 | 1.89 | 0.10 | 435.13 | 216.56 | 39.26 | -2.46 | -0.74 | 0.3 | 1.06 | 3.1 | 2.62 |
| A13 | O | 49.56 | 7.56 | 1.75 | 1.86 | 0.10 | 1611.53 | 309.53 | 40.87 | -11.49 | -12.81 | 1.14 | 1.67 | 11.55 | 12.48 |
| C22 | O | 69.65 | 7.30 | 3.18 | 1.84 | 0.06 | 1225.71 | 229.6 | 36.65 | -1.43 | -1.77 | 1.63 | 1.58 | 2.35 | 2.43 |
| C25 | O | 44.90 | 7.26 | 2.20 | 2.21 | 0.09 | 938.25 | 234.5 | 37.54 | -1.61 | -3.78 | 1.25 | 2.18 | 3.07 | 4.42 |
| A14 | O | 59.19 | 7.21 | 2.32 | 2.36 | 0.17 | 877.17 | 259.56 | 32.32 | -4.07 | -4.54 | 2.75 | 3.18 | 5.81 | 5.9 |
| C24 | O | 41.59 | 7.17 | 1.84 | 2.48 | 0.09 | 1132.19 | 282.22 | 38.71 | -5.53 | -5.95 | 0.97 | 1.62 | 5.71 | 6.18 |
| B21 | O | 55.01 | 6.92 | 2.50 | 2.54 | 0.12 | 633.26 | 205.06 | 39.12 | -3.14 | -5.18 | 1.67 | 2.73 | 3.77 | 6.49 |
| B22 | O | 69.93 | 6.31 | 1.34 | 1.34 | 0.09 | 1093.31 | 211.7 | 50.19 | -1.97 | -4.59 | 1.05 | 1.77 | 3.73 | 5.07 |
| C23 | O | 85.14 | 6.29 | 1.96 | 2.23 | 0.09 | 1111.04 | 183.48 | 38.48 | -3.86 | -5.13 | 2.21 | 2.45 | 4.89 | 4.87 |
| B19 | O | 49.20 | 5.80 | 2.39 | 1.93 | 0.08 | 2236.20 | 234.91 | 39.68 | -1.37 | -3.34 | 0.83 | 1.4 | 1.6 | 3.65 |
| A16 | O | 70.45 | 5.58 | 2.01 | 1.87 | 0.05 | 1198.43 | 253.04 | 42.97 | -2.53 | -5.24 | 1.22 | 1.44 | 3.04 | 4.68 |
| C20 | O | 91.36 | 5.10 | 1.78 | 1.69 | 0.09 | 1729.23 | 351.69 | 37.39 | -2.32 | -3.68 | 1.64 | 1.82 | 3.23 | 2.99 |
| B18 | O | 54.42 | 4.86 | 1.36 | 1.78 | 0.13 | 1025.16 | 272.76 | 40.57 | -0.77 | 0.85 | 1.01 | 0.99 | 1.28 | 1.97 |
| A19 | N | 42.61 | 8.93 | 2.67 | 2.91 | 0.16 | 1246.13 | 222.45 | 62.07 | -2.32 | -5.01 | 1.22 | 1.97 | 2.67 | 3.8 |
| B29 | N | 93.83 | 8.70 | 2.94 | 2.59 | 0.13 | 1398.73 | 196.57 | 62.44 | -5.54 | -7.87 | 1.88 | 2.72 | 5.87 | 8.5 |
| B25 | N | 85.45 | 8.35 | 2.98 | 2.49 | 0.17 | 68.93 | 94.12 | 52.39 | -5.69 | -6.73 | 2.06 | 2.95 | 6.06 | 7.4 |
| B24 | N | 84.50 | 8.12 | 3.15 | 2.99 | 0.15 | 791.76 | 127.8 | 42.56 | -6.79 | -15.7 | 2.8 | 3.83 | 7.81 | 9.58 |
| B26 | N | 86.30 | 7.01 | 2.48 | 2.24 | 0.12 | 97.82 | 157.56 | 55.43 | -3.12 | -5.4 | 1.97 | 2 | 3.72 | 6.22 |
| A18 | N | 71.61 | 6.97 | 2.47 | 1.67 | 0.08 | 562.50 | 358.56 | 41.28 | -1.45 | -2.42 | 0.46 | 0.48 | 1.56 | 3.55 |
| B27 | N | 82.64 | 6.48 | 2.25 | 1.88 | 0.16 | 433.91 | 103.1 | 49.44 | -2.06 | -2.88 | 1.69 | 3.16 | 3.36 | 5.3 |
| C28 | N | 69.21 | 6.29 | 2.18 | 1.99 | 0.16 | 2396.44 | 112.84 | 46.26 | -3.33 | -6.19 | 1.25 | 1.95 | 3.61 | 6.51 |
| C27 | N | 99.00 | 6.23 | 2.11 | 1.62 | 0.14 | 452.16 | 232.57 | 65.14 | -2.81 | -4.77 | 1.25 | 1.93 | 3.97 | 6.23 |
| A22 | N | 66.81 | 5.68 | 2.18 | 1.88 | 0.19 | 455.30 | 178.11 | 43.94 | -3.52 | -6.73 | 1.23 | 2.34 | 3.96 | 4.96 |
| A21 | N | 89.03 | 5.46 | 1.35 | 1.62 | 0.07 | 430.44 | 185.61 | 57.16 | -1.94 | -1.75 | 1.11 | 1.15 | 2.77 | 2.26 |
| C31 | N | 119.23 | 5.43 | 1.58 | 1.67 | 0.07 | 807.51 | 261.2 | 57.34 | -3.27 | -6.48 | 1.11 | 1.28 | 3.61 | 6.63 |
| B28 | N | 90.57 | 5.37 | 1.48 | 1.59 | 0.12 | 470.64 | 156.6 | 53.65 | -3.11 | -3.67 | 2.07 | 2.67 | 3.94 | 4.58 |
| A17 | N | 99.11 | 5.33 | 1.55 | 1.62 | 0.08 | 330.61 | 139.26 | 68.26 | -1.89 | -4.96 | 0.68 | 1.3 | 2.13 | 5.27 |
| C26 | N | 75.16 | 5.31 | 1.30 | 1.42 | 0.09 | 665.59 | 217.89 | 61.52 | -2.89 | 0.4 | 1.8 | 1.9 | 3.49 | 4.1 |
| C30 | N | 75.41 | 4.96 | 1.26 | 1.38 | 0.09 | 948.15 | 194.84 | 69.41 | -1.81 | -1.5 | -0.18 | -0.04 | 1.9 | 2.55 |
| C29 | N | 62.68 | 4.79 | 1.83 | 1.45 | 0.12 | 645.02 | 137.03 | 44.81 | -3.56 | 2.29 | 1.02 | 0.8 | 4.28 | 2.39 |
| A20 | N | 67.68 | 4.27 | 1.20 | 1.16 | 0.10 | 1299.92 | 136.85 | 54.39 | -0.69 | 0.39 | 1.45 | 1.13 | 2.53 | 3.15 |
| *Notes*: Sample codes are unique to each sample; the letter indicates the field harvested from. S: September, O: October, N: November, TPC: total phenolic content (g kg^-1^ ), CGA: chlorogenic acid (g kg^-1^ ), DiCQA: dicaffeoylquinic acid isomer (g kg^-1^), A7G: apigenin-7-*O*-glucuronide (g kg^-1^), PPO: polyphenol oxidase activity (10^3^ U g^-1^), Weight: inflorescence weight (g), EP%: edible portion as a percentage of the inflorescence weight. Samples are ordered by harvest and from highest to lowest TPC. | | | | | | | | | | | | | | | |

**2.8 Video collation of artichoke EP browning progression**

Supplementary Video 1. Screen shot from video collation of PPO-mediated browning of artichoke quarters taken over 90 min. The graphs from left to right show the change in CIE *L**, CIE *a**, CIE *b**, and CIE Δ*E** (dE*). Each line represents a harvest (early, mid, and late). Time points are the mean value of samples from that harvest (*n* = 18). Error bars at each time point indicate the standard error The video can be viewed by double clicking the .mp4 link below.

**2.9 Change in colour of EP during browning grouped by harvest**

Supplementary Figure 9. The change (∆) in colour parameters over time compared to the initial time point (0.5 min). A) ∆*L**, B) ∆*a**, and C) ∆*b**. Colour measurements taken at intervals between 0.5 min and 90 min after cutting the artichoke edible portion. Each line represents a harvest (September, October, and November). Time points are the mean value of samples from that harvest (*n* = 18). Error bars at each time point indicate the standard error.

**2.10 CIE *b** values of EP immediately after cutting grouped by harvest**

Supplementary Figure 10. CIE *b** values measured at 0.5 min compared between harvests (*n* = 18). Significance of Tukey HSD post-hoc test between groups indicated by bars above (*p* < 0.05). Boxes show the 25^th^ and 75^th^ quartiles with the median value indicated by the internal black line. The mean value is indicated by the white diamond. The vertical bars identify the maximum and minimum values excluding outliers. Individual data points are presented as black circles.

**2.11 Correlation between CIE *b** values of EP immediately after cutting and phenolics**

Supplementary Figure 11. Correlations between CIE *b** values at 0.5 min after cutting and phenolics. A) Chlorogenic acid concentration (g kg^-1^), B) Dicaffeoylquinic acid isomer (g kg^-1^), and C) Total phenolic content (g kg^-1^) (*n* = 54). Concentrations are presented per kilogram of edible portion fresh weight. Total phenolic content is presented as CGA equivalents. *R* = Spearman’s rank-order correlation coefficient (*r*_s_), with the corresponding *p* value. Trendlines were generated from linear regression and indicate the direction of association.

**2.12 Correlations between CIE colour parameters and phenolics 9 min after cutting**

Supplementary Figure 12. Correlations of chlorogenic acid concentration (g kg^-1^), dicaffeoylquinic acid isomer (g kg^-1^), and total phenolic content (g kg^-1^) with ∆*L**, ∆*a**, and ∆*E** values at nine min (*n* = 54). Concentrations are presented per kilogram of edible portion fresh weight. Total phenolic content is presented as CGA equivalents. The letter d is used to denote ∆ on the y-axis. *R* = Spearman’s rank-order correlation coefficient (*r*_s_), with the corresponding *p* value.

**2.13 Correlations between CIE colour parameters and phenolics 90 min after cutting**

Supplementary Figure 13. Correlations of chlorogenic acid concentration (g kg^-1^), dicaffeoylquinic acid isomer (g kg^-1^), and total phenolic content (g kg^-1^) with ∆*L**, ∆*a**, and ∆*E** values at 90 min (*n* = 54). Concentrations are presented per kilogram of edible portion fresh weight. Total phenolic content is presented as CGA equivalents. The letter d is used to denote ∆ on the y-axis. *R* = Spearman’s rank-order correlation coefficient (*r*_s_), with the corresponding *p* value.

**2.14 Yellow colour of EP phenolic extracts**

Supplementary Figure 14. Photo of three phenolic extracts from artichoke edible portions in 70% methanol + 0.1% formic acid. The methanolic extracts exhibit a strong yellow colour.
